# Examining acculturation in mixed-couples to test cultural transmission mechanisms

**DOI:** 10.1371/journal.pone.0266229

**Published:** 2022-04-06

**Authors:** Bernardo Guerra Machado, Roger Giner-Sorolla

**Affiliations:** 1 School of Anthropology and Conservation, University of Kent, Canterbury, United Kingdom; 2 School of Psychology, University of Kent, Canterbury, United Kingdom; Polytechnic Institute of Coimbra: Instituto Politecnico de Coimbra, PORTUGAL

## Abstract

The project sought to understand the factors which underlie cultural transmission, adapting self-reported methods from cross-cultural psychology and sociology to test the external validity of several constructs from existing evolutionary models. The target population were native-foreigner mixed-couples, allowing the analyses to benefit from asymmetrical cultural inputs. Sampling took place in Italy and Portugal, with recruitment relying on social networks, online newspapers, friends, organizations, universities, parishes, and embassies. The questionnaire was personally delivered or filled online. The validated variables were: contact with a population in which the majority endorses the culture being acquired, the relative quantity of friends from that culture, the perceived relationship quality with the companion, affective ties with one’s own family, and the desire and emotional components behind the culture-transmission motive (a concept similar to cultural conservatism). An unexpected strong, positive association between both cultural identities was found. Thus, it was suggested that these participants adopted an integrative orientation, allowing both cultural identities to blend, whereas most research so far focuses on assimilation scenarios. Overall, acculturation was driven by either conformity to the majority or random learning, without discarding the influence of preferred demonstrators, and the emotional bounds embedded in the individual’s cultural identity. Acculturation proved to be flexible and potentially changing according to the cultural trait being examined.

## Introduction

### Cultural learning and the psychology of acculturation

Studying acculturation is relevant to the gene-culture coevolution perspective, to better understand the mechanisms behind cumulative cultural learning [1]. Research within this field relies mostly on theoretical models adapted from population genetics and derived from anthropological data, laboratory experiments [2], and economic games [3]. In their turn, cross-cultural psychologists and sociologists have been studying the acculturation process in several groups of migrants–defining it as the changes motivated by continuous contact with individuals from a different culture, comprising aspects such as attitudes, behaviours, values, and sense of cultural identity [4]. Furthermore, several acculturation orientations have been theorized [5], two being particularly relevant to the present context: assimilation, when the culture being acquired substitutes the former one; and integration, when both cultural identities coexist. The latter has been documented to be the most efficient one in terms of coping with the psychological challenges posed by the acculturation experience, designated as *acculturative stress* [6]. The methods employed by cultural psychology and sociology usually consist of interviews and self-reported measures. Age at arrival, length of stay, socioeconomic status, education, and language proficiency [7] are among the most documented predictors of acculturation. However, these works seldom seek to address questions of evolutionary nature. In parallel, their findings and methods only with few exceptions [8] have been considered when studying cumulative cultural learning. The present study also intends to highlight the scientific interest of bringing these two approaches closer. Several theoretical models of cultural transmission within the migratory context will be presented. Then, the external validity of the factors involved, meaning their relation with acculturation, will be tested using self-reported measures.

### Cultural identities

The *heritage culture* is defined as the predominant culture in which an individual was raised. In the present study, focused on mixed-couples, c*ultural maintenance* refers to the degree of identification with the heritage culture; whereas *acculturation* concerns the identification with the companion’s culture. Several studies have shown that these constructs are approximately independent and should be both assessed [4]. This has been supported by cognitive studies aiming to dissect *bicultural frame switching* [9]–the capacity to store two behavioural patterns, ready to respond to distinct cultural primes.

### Hypotheses (H) and predictions (P)

Previous research has pointed out that the null model of cultural transmission should be *random copying*, the simplest way through which the dispersion of cultural variants can be explained [10]. Thus,

*H0: Acculturation occurs by blind imitation of the cultural variants available in a certain population*.

This mechanism should not be confused with conformist learning [11]–a bias leading us to prefer the commonest traits within a certain population, which can theoretically optimize adaptation to changing environments [12, 13]. Mesoudi [14] designed a model in which between-groups cultural variation could be maintained, despite migration, if individuals who arrive in a new group are biased to conform to the rule of the majority. The model then assumed that acculturation is impaired by assortation (here referred as pair assortation), the preferential contact with individuals who share the heritage culture. Therefore, it is hypothesized that

*Ha: Acculturation mainly relies on conformity to the majority, just as long as individuals from different groups interact with those from the local dominant culture*.

Pa1: Foreigners will report higher levels of acculturation than natives;

Pa2: Natives that never had direct contact with the companion’s country of origin will have null levels of acculturation;

Pa3: Pair assortation impairs acculturation.

Another model proposed by Mesoudi [14] assumed that, together with conformity, a payoff-biased social learning could be involved–a heuristic mechanism which adopts the attributes perceived as being the most beneficial. This mechanism will translate into the avoidance of social punishment by embracing the local norms. In this context, Bunce and McElreath’s [15] model of minority cultures’ conservation is conceptually useful, as it depicts acculturation as a process of negotiating norms, enhancing inter-ethnic coordination. Erten et al.’s [16] acculturation orientation and cultural evolution model elucidates which individual constructs can potentially affect such normative alignment: cultural conservatism and the willingness to interact with people from other cultures. The former construct will be taken as equivalent to the culture-transmission motive (CTM). This concept has a previously validated measurement, and is defined as the appreciation for one’s culture, stable across time and generations, alongside with the motivation to preserve and pass it to the descendants [17]. The willingness to interact with foreigners will be assumed to be underlined by both pair and normative assortation–the conscious preference to interact according to the norms of one’s heritage culture. Hence,

*Hb: Acculturation is payoff-driven. Individuals from different cultural groups seek to enhance coordination in a negotiation-like process of social norms, which is affected by CTM and both forms of assortation*.

Pb1: Payoff-biased social learning will positively associate with acculturation;

Pb2: Low levels of pair and normative assortation will be associated with higher acculturation scores;

Pb3: Payoff-biased social learning and both pair and normative assortation should be negatively related.

Pb4: CTM will be positively related with cultural maintenance, and it can be either negatively correlated with acculturation or independent, depending if the orientation is, respectively, assimilative or integrative (assuming those who form mixed-culture couples would be high on the spectrum of willingness to interact with foreigners);

Another possibility is that cultural transmission relies on *model learning*, being biased towards preferred demonstrators–the people we opt to learn from, based on certain cues [18, 19]. If so, emotions should play a relevant role in both acculturation and cultural maintenance. The current literature has focused mainly on mixed marriages’ predictors [20], which curiously include feelings of affection towards one’s family [21]. Because it can be argued that one’s companion is a favoured model, the perceived relationship quality (PRQ) may function as a proxy to measure the affective bonds’ weight on acculturation. Additionally, acculturation should require the development of a vast network of relationships with demonstrators from the culture being acquired–tested through the relative quantity of close friends from that culture. Nevertheless, emotional aspects might also include ties between the individual and their own culture (CTM-emotion), which could affect both cultural maintenance and acculturation. Thus,

*Hc: Acculturation is determined by model learning, and thereby explained by preferred demonstrators and emotional bonds*.

Pc1: PRQ is positively related with acculturation, trust and commitment being the components arguably more connected with the idea of a preferred demonstrator;

Pc2: regarding friendship networks, acculturation will be particularly associated with the relative quantity of friends from the companion’s culture;

Pc3: rather than the time of contact with the culture being acquired, acculturation should be predicted by the time spent with the companion;

Pc4: because it is a predictor of mixed marriage, positive feelings of affection with one’s own family should not only be positively related with cultural maintenance, but also with acculturation;

Pc5: CTM-emotion should be related with both cultural maintenance and acculturation.

The final hypothesis to be advanced is, that without any markedly dominant mechanism,

*Hd: Acculturation recruits several different types of social learning*.

Pd1: all or several of the previous hypotheses will receive partial support;

Pd2: natives and foreigners will potentially show different acculturation patterns.

## Materials and methods

### Ethics statement

The present research has been approved by the School of Anthropology and Conservation’s Research Ethics Committee, at the University of Kent. All the participants who took part in it have signed an informed consent.

### The target population

In native-foreigner mixed-couples, there is a particular two-way process of cultural exchange. Natives may have had little or no contact with populations in which the majority belongs to the foreign culture of the companion, therefore conformity and payoff-biased social learning can be minimal for them. Conversely, foreigners should be fully experiencing these aspects. Such asymmetrical inputs provide a unique ground to test the listed mechanisms. No prior study could be found seeking to benefit from this. Italy was the primary target for collection of data because migrants represent around 9% of the Italian population, and tend to opt for an integrative cultural orientation [22]. In fact, intermarriages including one native represent around 10% of its total marriages [23]. Another reason sustaining the sampling choice, was Italy’s considerable within-country cultural heterogeneity, which is expected to boost the generalisation of this study’s results. In order to attain a larger sample size, the efforts to recruit participants included a second country. Portugal was chosen, as its immigrant population is predominantly preferential to an integrative orientation as well, and the majority have origins from outside the European Union [24], potentially increasing the sample’s cultural diversity and, again, the generalisation of the results. Finally, Portugal and Italy have a solid cultural proximity, allowing to merge the sample without raising statistical noise–as these countries share an extensive common cultural past under Roman rule, engraved in deep linguistic and institutional similarities, which were reinforced by long trading relations.

### Questionnaire translation

The questionnaire was first written in English (S1 Text), and later translated by the lead researcher to Portuguese (S2 Text) and, with the help of a professional translator, to Italian (S3 Text). Measurement invariance was verified before merging both data sets (S1 Table).

### Variables and measurements

- Demographic data: potentially useful as control variables, included sex, age, subjective socioeconomic condition (SEC), age at arrival (exclusively for foreigners), and number of children (total and from the present partner).
- Contact: measured by asking natives the total number of months spent in the companion’s country of origin; and foreigners how many years have they spent in Italy/Portugal.
- Affective ties with family: participants were asked to rate their overall relationship with their parents and close family using a 6-point Likert scale.
- Language proficiency: measured by self-evaluating both the capacity to understand and to express through the language of their companion, from 0 to 100%, then calculating the mean. This variable will not be considered a predictor of acculturation, since it is a cultural trait, but can be useful to run parallel analyses to the general acculturation score.
- Cultural maintenance and acculturation: these measurements relied on the Vancouver Index of Acculturation [4], excluding the items “I am willing to marry a person from other/my heritage culture”. Following recommendations by Lopez-Class *et al*. [25], they were revalidated for the present population (S1 Appendix). Cronbach’s α = .90 (cultural maintenance) and .89 (acculturation).
- Perceived relationship quality: the PRQ scale [26] was used, removing the passion component to avoid being invasive. The 5 *quasi-*independent components left were satisfaction, commitment, intimacy, trust, and love; each presenting 3 items. For the measurement to fit, the errors of each set of items corresponding to a components were not considered independent; which implied calculating the score on AMOS (S1 Appendix). The duration of the relation was also assessed.
- Culture-transmission motive: measured by adapting Mchitarjan and Reisenzein’s scale [27], having three components–the desire to maintain and transmit the culture of origin (CTM-desire), the emotions associated with its loss and media reports regarding it (CTM-emotion), and action tendencies aiming to preserve it (CTM-action). The latter was dropped (S1 Appendix). The questions referring to marital preferences were simplified to “my family would prefer that I married someone from my culture of origin.” Cronbach’s α = .85 (desire) and .79 (emotion).
- Pair assortation: obtained by asking the number of close friends from one’s heritage culture, from their companion’s culture, and from third other cultures. Then, the relative quantity of each category was estimated, with pair assortation referring to the portion of friends sharing their own culture.
- Normative assortation: applied only to foreigners. A novel measurement with four items was proposed, seeking to quantify the participant’s preference for social norms from their heritage culture. Questions were rated using a 5-point Likert scale in terms of agreement-disagreement. In the end, only the marker item was used because the scale’s reliability was low (S1 Appendix). Cronbach’s α = .56.
- Payoff-biased social learning: was exclusive to foreigners. This new measurement aimed to establish a score for the perceived benefit of adapting to the local culture. It assumes that, during the initial period of adaptation, to the reported levels of social disapproval due to cultural differences corresponds the perceived acculturation’s payoffs, to avoid peer sanctions. The number of items and the Likert scale used were the same as in the previous measurement. Cronbach’s α = .77.
- Finally, participants were asked to rate, in a 7-point Likert scale, how well they understood the questionnaire (S1 Text).

### Participants

The couples were composed of a native, educated within the Italian/Portuguese culture, and a foreigner, raised in a different country and cultural background. Each couple was currently living in Italy/Portugal, and had done so for most of their relationship. The report of deviant cases excluded and the ones included can be seen in S2 Table and S3 Table, respectively. Recruitment was made through social networks, online newspapers, friends, organizations, universities, parishes, and embassies. When possible, the questionnaire was personally delivered, seeking to increase the anthropological value of the present study. It was frequent for participants to share relevant information during conversations. However, due to resources constraints, data collection had to partially rely on online questionnaire filling through Google Forms. From the original set of 163 participants, 19 were excluded, leaving a sample size of 144 (S1 Dataset). From these, 86 (41 foreigners and 45 natives) were living in Italy, and 58 (31 foreigners and 27 natives) in Portugal. Descriptive statistics for both groups can be seen in Table 1.

**Table 1:** Descriptive analysis of participants from Portugal and Italy.

	Portugal (N = 58)	Italy (N = 86)
Sex, male (%)	24/58 (41.4)	37/86 (43.0)
Age in years, median (IQR)	53.0 (42.8–57.0)	40.5 (29.8–52.3)
Years of education, mean (SD)	18.1 (4.5)	17.0 (3.8)
Years in Italy/Portugal, median (IQR)	24.0 (13.8–24.0)	13.5 (5.3–29.0)
Total number of months spent in the foreign companion’s country, median (IQR)	12.0 (2.8–51.0)	2.0 (0.0–22.0)
Duration of the relation in years, median (IQR)	20.0 (13.0–31.0)	8.5 (3.5–16.0)
Subjective socioeconomic condition, mean (SD)†	3.9 (0.8)	3.7 (0.7)
Questionnaire comprehension, mean (SD)‡	6.2 (0.9)	6.1 (1.0)

Discontinuous variables are expressed as number (%). SD, standard deviation. IQR, interquartile range. †Assessed with the question “How well do you live with your present household income?” in a 5-point Likert scale (higher values representing greater perceived socioeconomic condition).

‡ Assessed with the question “How well did you understand the questions in this survey?” in a 7-point Likert scale (higher values representing better understanding).

### Data analyses

The data were analysed using IBM SPSS Statistics, version 25. With the exception of PQR, scale scores were obtained through factor analysis, using Unweighted Least Squares for the extraction, as distributions were not normal. The few cases exhibiting a normal distribution were registered in S4 Table. Scores were generated for each group (natives/foreigners). Mediation analyses relied on the INDIRECT macro [28], version 4.1, performing 5000 bootstrapping resamples. The native and the foreigner samples were assumed to be independent, for several reasons. First, when the questionnaire was personally delivered, the participants were asked not to exchange opinions during its filling, which was supervised. In the online questionnaire, it was stated that the participants should fill the questionnaire individually. This helped assuring the answers were not conditioned between partners. Second, the present study assumes, and aims to highlight, that natives and foreigners have distinct cultural inputs and motivations to acculturate. Also, because of this assumption, not the exact same set constructs was assessed for each group, producing some results impossible to match. Finally, there were cases in which only one member of the couple participated, augmenting the number of unpaired data points.

The power of the analyses was calculated using G*Power, version 3.0.10. A previous meta-analysis indicated that the size of the effects relating psychological constructs with acculturation tends to be of .16 [29]. That would imply a sample of 304 individuals to attain 80% of power. However, given the exploratory character of the present study, a smaller sample was considered enough. This way, the novel scales could be tested, the innovative aspects of the methodology could be shared with the scientific community, and constructive critics be gathered. In the future, an even more solid approach can be designed, justifying the gathering of larger samples. Nevertheless, the present study can assure safe ground for significate effects with size .23 or larger, when considering the full sample; and size .32 or larger, when using the native/foreigner samples. For an effect size of .16 the full sample presents a power of 48%, whilst the native/foreigner samples have a power of 27%. Table 2 presents the estimated power for each of the main analyses.

**Table 2:** Estimated power for each of the main analyses.

	Natives	Foreigners	Full sample
Contact effect (yes/no)	.55	-	-
Time of contact	.28	.44	-
Pair assortation	.10	.33	.26
Payoff-biased social learning	-	.07	-
Normative assortation	-	.05	-
CTM-desire	1.00*	.99*	1.00*
CTM-emotion	.92*	.99*	1.00*
Perceived relationship quality	.68	.25	.84*
Relative quantity of friends from the companion’s culture	.33	.37	.89*
Time spent with the companion	.33	.22	.54
Affective ties with the own family	.47	.81*	.89*

Results in which the recommended 80% power was attained were marked with an *.

Regarding outliers, the data obtained was substantially clean (S1 Fig and S2 Fig). Generally, there were no marked violations of the assumption of homoscedasticity, concerning the main linear regressions run (S3 Fig and S4 Fig).

## Results

### Preliminary analysis

In order to check which demographic data should be controlled for, a correlation analysis was run. Those significantly related with acculturation or cultural maintenance were to be used as control variables in the tests to come. Results are shown in Table 3.

**Table 3:** Pearson’s correlation of cultural maintenance and acculturation with potential control variables.

		Acculturation	Age	Years of education	Subjective SEC	Age at arrival	Number of children
Cultural maintenance	r	.62**	.16	-.02	.19*	.19	-.01
	N	143	143	143	143	69	142
Acculturation	r		.13	-.12	.15	-.13	-.02
	N		144	144	144	70	143

Significant results (p < .05) are signalled with *, and highly significant (p < .001) with **.

**Fig 1:**
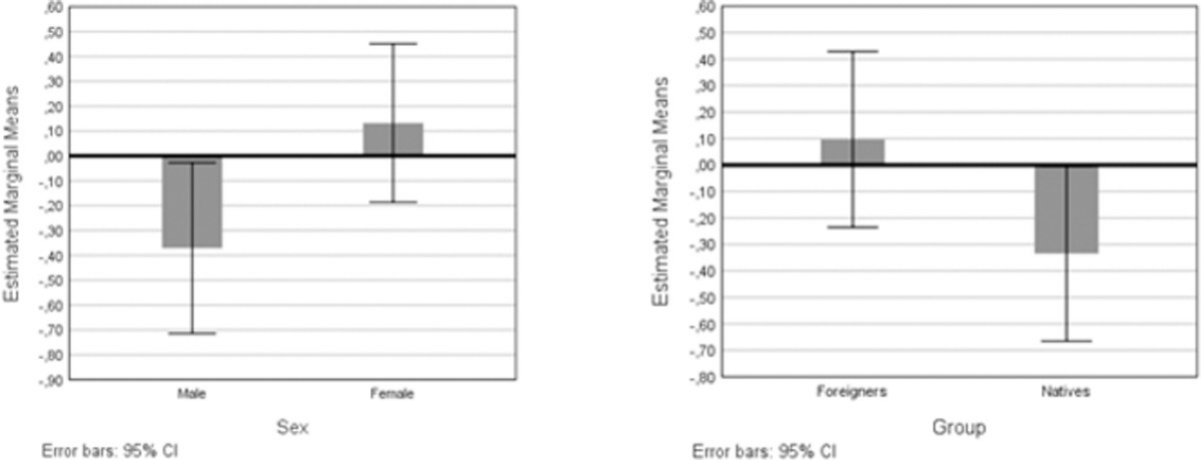
Estimated marginal means for the acculturation score according to sex (left) and group (right).

Through a General Linear Model (GLM), the effects of sample provenance (Italy/Portugal), type of participation (online/interview), gender, group (native/foreigner), and the respective interactions on cultural maintenance and acculturation, were assessed. Some interactions could not be performed, because only one couple from Portugal was interviewed, while the rest used the online questionnaire. According to the results presented in Table 4, merging the Italian and the Portuguese samples does not raise noise for the analyses; there are acculturation differences due to sex, which require examination; and natives and foreigners should be studied in separate analyses. The interaction type of participation*sex was significant in cultural maintenance, with females responding to the online questionnaire having a greater average score. However, the main effects were non-significant and all the marginal means’ 95% confidence intervals still overlap (not shown). From these two predictors, only sex affected the acculturation scores, therefore, it can be advocated that this interaction may have resulted from convergent fluctuations of the two variables and there is no systematic bias associated with the type of participation. Examining Fig 1, it becomes clear that women and foreigners tend to exhibit higher acculturation scores.

**Table 4:** GLM results testing the effects of sample provenance (Italian/Portuguese), type of participation (online/interview), sex, group (native/foreigner), and the interactions on cultural maintenance and acculturation.

	Cultural maintenance			Acculturation		
Model and predictors	num. df	F	η_p_^2^	num. df	F	η_p_^2^
Corrected Model	14	1.49		13	1.35	
Sample (Italian/Portuguese)	1	.34	.003	1	.35	.003
Participation (online/interview)	1	1.32	.010	1	.52	.004
Sex	1	2.20	.017	1	6.69	.049*
Group (native/foreigner)	1	.19	.002	1	5.27	.039*
Sample*Participation	1	.90	.007	1	.93	.007
Sample*Sex	1	.73	.006	1	.23	.002
Sample*Group	1	.37	.003	1	.05	.000
Participation*Sex	1	4.84	.036*	1	1.40	.011
Participation*Group	1	.05	.000	1	.03	.000
Sex*Group	1	.30	.000	1	1.26	.010
Sample*Sex* Group	1	.04	.000	1	.13	.001
Participation*Sex* Group	1	1.82	.014	1	.05	.000
	N = 143, R^2^ = .14, den. df = 128			N = 144, R^2^ = .12, den. df = 130		

Significant results (p < .05) are signalled with

*. Covariates: subjective SEC (exclusively for cultural maintenance).

### Hypothesis A

**Fig 2:**
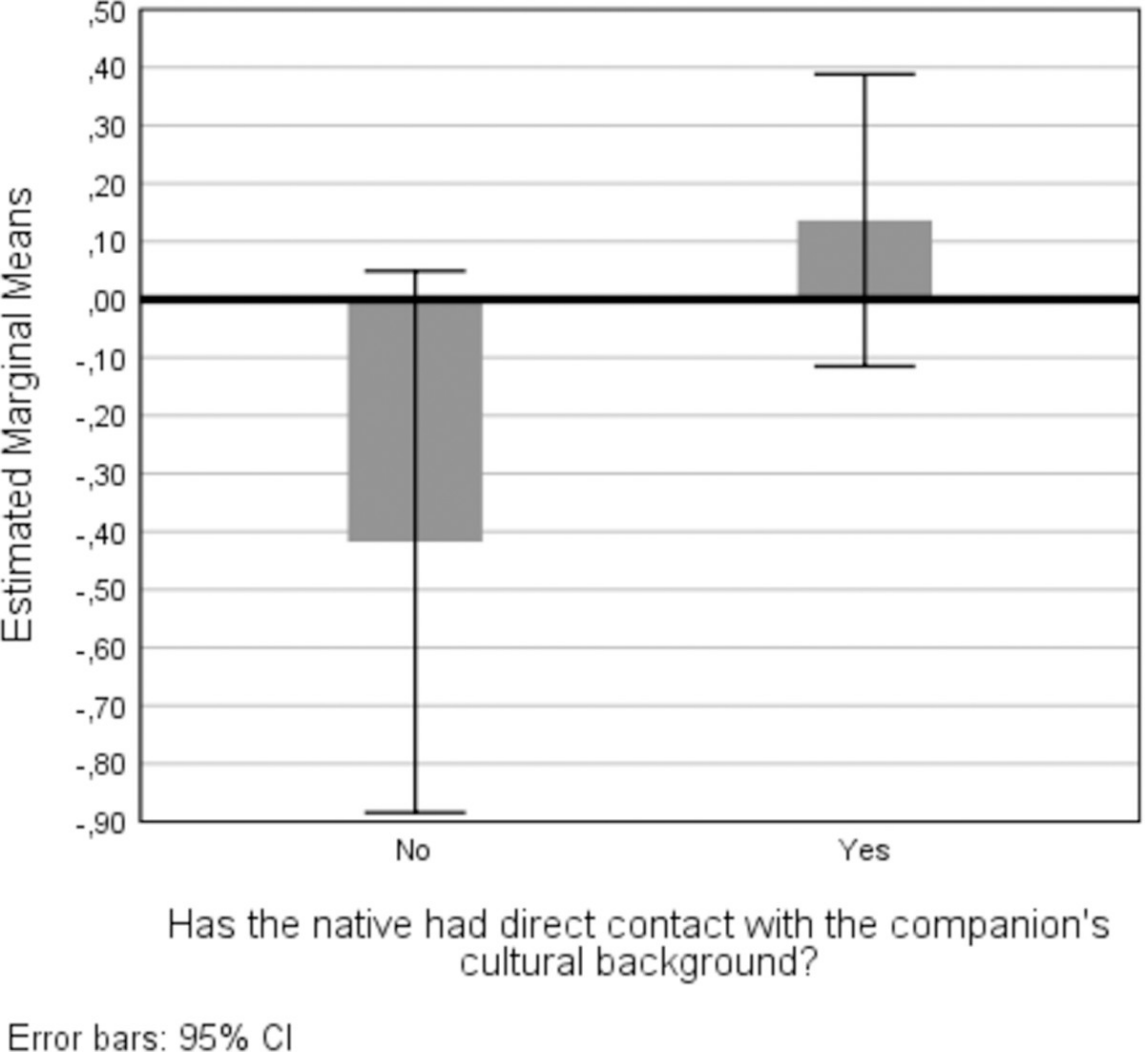
Estimated marginal means for the native’s acculturation score depending on contact.

It was observed that foreigners tend to score higher on acculturation, supporting Pa1. In the native sample, the contact and no-contact groups were compared through a GLM. A medium effect: *F*(1,69) = 4.35, *p* = .04, *η*_*p*_^*2*^ = .059 was obtained (Fig 2). When the comparison was balanced using alternative categories–“scarce contact” (until four months of contact, *N* = 37) and “high contact” (more than four months, *N* = 34)–no significant difference emerged: *F*(1, 69) = 1.44, *p* = .23, *η*_*p*_^*2*^ = .02. The same occurred when running a bivariate regression with the total number of months of contact predicting acculturation: *β* = .16, *p* = .18. Contrarily, a marginal significance was attained testing the number of years foreigners spent in Italy/Portugal: *β* = .21, *p* = .08.

Pa2 was not supported, because the natives who never had direct contact with the companion’s country of origin had, nonetheless, positive acculturation scores: *N* = 16, $\bar{X}$ = 5.56 (above the scale midpoint), *SD* = 1.19. Similarly, Pa3 was not met, providing that there was no significant, negative relation between pair assortation and acculturation in natives, foreigners, or jointly: *β* = .08, *p* = .49; *β* = -.18, *p* = .15; and *β* = -.11, *p* = .19; respectively.

### Hypothesis B

In order to check if social learning is biased towards the adoption of the most beneficial traits, a bivariate regression with the payoff-biased social learning score predicting acculturation was run: *β* = -.05, *p* = .69; with no support being obtained for Pb1. There was no significant association between acculturation and both pair (see Ha) and normative assortation: *β* = -.02, *p* = .86; contrary to what was stated in Pb2. Additionally, payoff-biased social learning did not correlate with normative assortation: *r*(70) = .17, *p* = .15; contradicting Pb3. Several regression models are presented on Table 5, using CTM-desire and CTM-emotion, either together or individually, to predict cultural maintenance and acculturation.

**Table 5:** Standardized coefficients of the CTM’s components predicting cultural maintenance and acculturation, together and in separate.

		Cultural maintenance	Acculturation
		β together (individually)	β together (individually)
Natives	CTM-desire	.59** (.64**)	.41* (.49**)
	CTM-emotion	.10 (.39*)	.17 (.37*)
Foreigners	CTM-desire	.81** (.76**)	.25† (.45**)
	CTM-emotion	.07 (.44**)	.21* (.41**)
Full sample	CTM-desire	.64** (.69**)	.36** (.47**)
	CTM-emotion	-.07 (.46**)	.30* (.47**)

Marginally significant results (p < .10) are signalled with †, significant (p < .05) with

*, and highly significant (p < .001) with

**. Numbers outside brackets refer to regressions considering both components. Inside brackets are the individual results. Control variables: subjective SEC (exclusively for cultural maintenance).

The CTM, especially the desire component, was positively related with cultural maintenance, supporting Pb4. However, the fact that at least one component had a positive association with acculturation did not met Pb4.

### Hypothesis C

After testing the general effect of PRQ using a bivariate regression, its components were used to perform a multiple regression predicting the acculturation score (Table 6), considering one item for each [26]. Only two aspects deviated from Pc1: the relation was not significant for foreigners (*β* = .15, *p* = .22), and the trust component was not underlying the effect.

**Table 6:** Standardized coefficients obtained regressing the perceived relationship quality, then its components individually, on acculturation.

	Natives	Foreigners	Full sample
	β	β	β
Perceived relationship quality	.28*	.15	.22*
Satisfaction	.56*	.19	.31*
Commitment	.34*	.28	.40**
Intimacy	-.12	-.17	-.18
Trust	-.23	-.13	-.13
Love	-.25	-.03	-.15

Significant results (p < .05) are signalled with

*, and highly significant (p < .001) with

**. The general effect was obtained running bivariate regressions, whereas the component’s integrated multiple regressions.

The relative quantity of close friends from the companion’s culture, in a bivariate regression, was significantly related with acculturation in the full sample (*β* = .26, *p* = .001), but not in the native (*β* = .18, *p* = .13) and the foreigner ones (*β* = .19, *p* = .11). Thus, the tests regarding Pc2 were inconclusive. Accordingly, the effect of the time spent with the companion on acculturation formulated in Pc3, was significant in the full sample (*β* = .17, *p* = .04), and not in the native (*β* = .18, *p* = .14) and foreigner (*β* = .14, *p* = .24) ones. Running another bivariate regression, the association between affective relations with one’s own family and acculturation was tested, being only marginally significant for natives (*β* = .22 *p* = .06), but significant for foreigners (*β* = .32, *p* = .01) and in the full sample (*β* = .26, *p* = .001), supporting Pc4. The corresponding effects, controlling for subjective SEC, on cultural maintenance produced significant results for natives (*β* = .26, *p* = .04) and in the full (*β* = .20, *p* = .03) sample, albeit not for foreigners (*β* = .14, *p* = .26). As seen in Table 5, CTM-emotion, regressed alone, was always significantly related with acculturation and cultural maintenance, in accordance with Pc5. It should be further noted that the foreigners’ acculturative advantage disappeared when the relative quantity of friends from the companion’s culture was included in the GLM as a covariate, contradicting Pa1: *F*(1, 139) = .84, *p* = .36, *η*_*p*_^*2*^ = .006, with a significant omnibus effect *F*(2, 139) = 5.67, *p* = .004, *R*^*2*^ = .075. Similarly, applying the same control in a regression model with the number of years spent in Italy/Portugal predicting the acculturation score, a non-significant result was produced: *β* = .17, *p* = .17. However, by extracting such variance, the native’s contact effect increased, *F*(1, 66) = 5.53, *p* = .02, *η*_*p*_^*2*^ = .077. In the scarce-high contact comparison, the lack of significance stood: *F*(1, 66) = .98, *p* = .33, *η*_*p*_^*2*^ = .015.

### Hypothesis D

As shown on Table 7, both Pd1 and Pd2 received support. Accordingly to the predictions, all other hypotheses received partial support. Furthermore, the results were not always parallel between natives and foreigners, particularly regarding the role of the PRQ, time of contact, affective ties with the family, and the CTM’s components.

**Table 7:** Representation of the results obtained for the other hypotheses, across samples.

		Natives	Foreigners	Full sample
Hypothesis A	Contact effect (yes/no)	1,2	-	-
	Time of contact	2	2	-
	Conformity	2	-	-
	Pair assortation (shared with Hb)		1	1,2
Hypothesis B	Payoff-biased social learning	-		-
	Normative assortation	-	1	-
	CTM-desire	2	2	2
	CTM-emotion (shared with Hc)	2	2	2
Hypothesis C	Perceived relationship quality	1,2		1,2
	Relative quantity of friends from the companion’s culture	2	1	1
	Time spent with the companion	2		
	Affective ties with one’s own family	2	2	2

Green sections: obtained support. Yellow sections: dubious results. Red sections: failure to reject the null hypothesis. 1: disparate results obtained in cultural maintenance (S5 Table), and 2: in language proficiency (S7 Table).

### *Post hoc* analyses

#### Variables associated with acculturation’s sex differences

In the preliminary analyses it was shown that the participants’ sex accounted for a significant amount of variance in acculturation (Table 4, Fig 1). Several GLMs were run with sex as the fixed factor and the remaining constructs as the dependent variable, to unravel which could account for the sex difference. CTM-emotion was the only candidate: *F*(2, 142) = 4.61, *p* = .03, *η*_*p*_^*2*^ = .031.

#### The positive correlation between the two cultural identities

**Fig 3:**
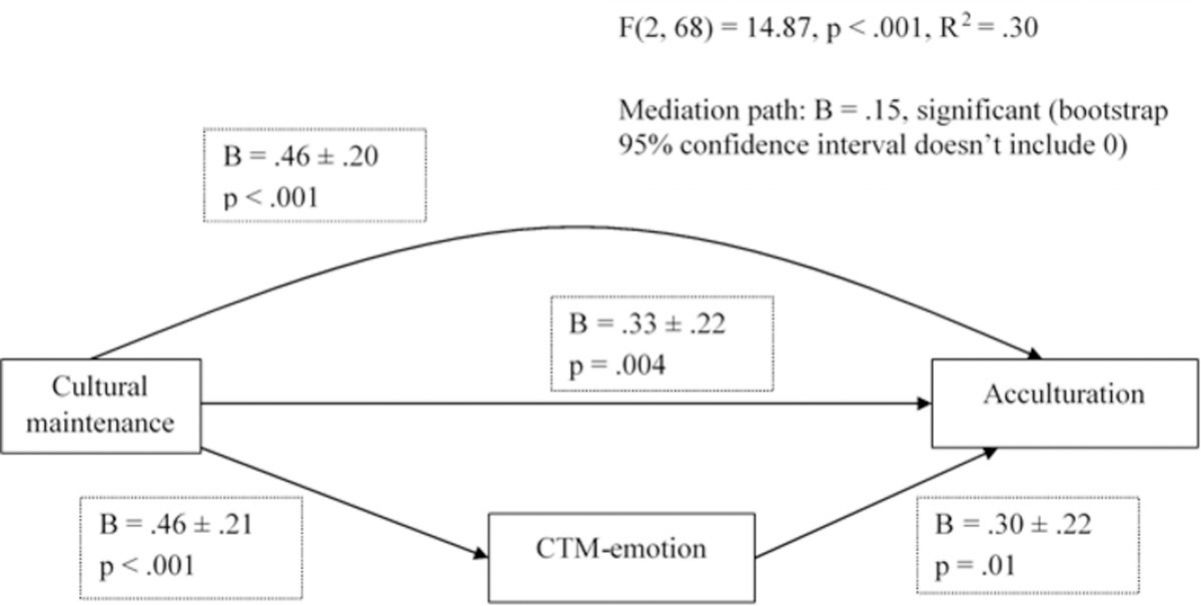
Representation of the partial mediating effect of CTM-emotion on the relation between cultural maintenance and acculturation obtained in the foreign sample. This pattern was also detected in the full sample, but the mediation effect was attenuated (not shown).

The association between cultural maintenance and acculturation remained when running separate bivariate correlations within the native and foreigner samples: *r*(70) = .75, *p* < .001; *r*(69) = .48, *p* < .001; respectively. Then, seeking to contextualize these results, a set of mediation analyses was performed. The relationship with one’s own family, the PRQ, and CTM-emotion were the chosen mediators. CTM-desire was excluded because, rather than being caused by cultural maintenance, it shares with it a relation of mutual reinforcement. The only significant model obtained is represented in Fig 3. Then, the link between CTM-desire and acculturation was suppressed running a multiple regression controlling for cultural maintenance: *β* = -.01, *p =* .95 (natives); *β* = .20, *p =* .23 (both predictors became non-significant for foreigners); *β* = .08, *p =* .41 (full sample).

#### The CTM’s relation with other variables

As it could be argued that pair and normative assortation are just consequences of CTM, their shared variance was examined in a set of bivariate regressions, using the full sample. CTM-desire was not a significant predictor of pair assortation (*β* =.-01, *p =* .89), but reached marginal significance with normative assortation (*β* = .23, *p =* 06); contrastingly, CTM-emotion predicted pair (*β* = .19, *p* = .02) but not normative assortation (*β* = .08, *p* = .52). Regarding foreigners, the link between CTM-emotion and pair assortation was lost (*β* = .09, *p =* .48). Additionally, one participant wondered if he would give the same scores to CTM’s questions if he had children. . . Answers are provided in Table 8.

**Table 8:** The most relevant results of the bivariate regressions using either the total number of children or the number of children with the current partner to predict the CTM components score.

	Natives	Foreigners	Full sample
Children with the current partner	CTM-emotion	-	CTM-emotion
			β = -.14†
	β = -.20†		
All children considered	CTM-desire	CTM-emotion	CTM-emotion
	β = -.20†	β = -.26*	β = -.23*

Marginally significant results (p < .10) are signalled with

† and significant (p < .05) with *.

#### The effect of the relative quantity of friends from third other cultures

To explore the relevance of the relative quantity of friends from third other cultures, two linear regressions were run. Results are shown on Table 9.

**Table 9:** Regression coefficients relating the relative quantity of friends from third other cultures with cultural maintenance and acculturation.

	Cultural maintenance	Acculturation
	β	β
Natives	-.26*	-.25*
Foreigners	.11	-.05
Full sample	-.09	-.20*

Significant results (p < .05) are signalled with *. Control variable: subjective SEC (exclusively for cultural maintenance).

#### Further analyses contained in the *Supporting information*

The Supporting information contains the predictors of cultural maintenance (S5 Table); its explained variance (S6 Table); parallel tests run on language proficiency (S7 Table and S8 Table); and the total variation explained in the acculturation scores (S9 Table). Cultural maintenance sometimes differed from what was previously observed: the PRQ produced non-significant results; normative and pair (though not for natives) assortation were marginally significant; the relative quantity of friends from the companion’s culture was negatively related with the foreigner’s cultural maintenance score. Similarly, language proficiency presented several distinct outcomes: total time of contact rather than the contact effect predicted the natives’ acculturation score; the time spent with the companion and the relative quantity of friends from the companion’s culture became significant in the native sample; finally, no significant effects were detected for PRQ, CTM, cultural maintenance, the affective ties with the own family, and the relative quantity of friends from third other cultures. Therefore, concerning this cultural trait, support was mostly given to Hc in the native sample, while foreigners produced inconclusive results.

## Discussion

It should be noted that this study referred to cultural transmission experienced by adults in the migratory context. Thereby, horizontal transmission (relative to peers of the same generation, related or not) is virtually the only form of cultural exchange being considered; whilst vertical and oblique transmission (resulting from interactions with individuals from older generations, of direct ascendancy or not, respectively) [30] are assumed to be residual. Next, it must be taken into account that some authors have hypothesised that some migrants may exhibit a *mobiocentric personality type* [31], a psychological inclination to be on the move. Such specificity would complicate the analyses by potentially biasing the foreigners’ behaviour. Nevertheless, anthropologists empirically challenge this view by documenting migration as a highly diverse and mutable phenomenon, to which a wide range of typologies are ascribed [25, 32]. It can be concluded that, while studying exclusively the process of horizontal transmission on adults, the results obtained here can be generalized, providing a reasonable contextualization.

Age at arrival and subjective SEC did not significantly influence acculturation, in contrast with several past works [7]. Interestingly, subjective SEC was positively correlated with cultural maintenance, suggesting high status individuals might be better able to keep a stronger tie with their heritage culture. Then, instead of approaching orthogonality, as in the original study validating the scale [4], cultural maintenance and acculturation were positively correlated, presenting a large effect size. Whilst the correlation was not novel, the direction of the effect was [33]. Contrary to the present study, past research may have mostly considered populations tendentially endorsing assimilative or segregated orientations, rather than the integrative one [5]–in which identification with both cultures can be positively related. The fact that cultural maintenance did not predict language proficiency, indicates that such association might be merely an artefact of the acculturation orientation. Still, it is possible that integrating a second culture can somehow reinforce identification with the heritage one; or that underlying both cultural identities there is a general appreciation for Human cultures. If so, Carlson & Güler’s [34] construct of *cultural involvement*, could well be adopted to measure the importance given to cultural aspects, globally. However, because it is based on very similar items to those used here to measure acculturation and cultural maintenance, a new set of questions should be advanced. It could substitute the CTM-emotion in the present context, but not without borrowing items from it, in order to propose a new scale of cultural involvement.

Alone, the fact that foreigners had on average higher acculturation scores fails to distinguish conformity from random learning. There are variables related with the other hypotheses which could be creating a confounding effect, such as the relative quantity of friends from other cultures (though it was only significant in the complete sample). The key point to sustain Ha was that natives who never had contact with their companion’s country of origin should have a null acculturation score. Despite having significantly lower scores than the contact group, they were above the scale’s midpoint. Interestingly, rather than time of contact, a rule of thumb contact-no contact seemed to be influencing acculturation, favouring the conformist learning scenario more than that of random transmission. This effect accounted for around 7% of the variance in the natives’ acculturation scores. Conversely, time of contact affected language proficiency (S7 Table). Indeed, two participants affirmed in their own way: “The fact that I also identify with my companion’s culture has more to do with the time I lived in his country than with the habits acquired from him”. Conformity possibly enhances acculturation, but no convincing evidence was obtained in the present study supporting its central role as assumed in Mesoudi’s model [14]. In turn, pair assortation can be seen as an indicator of segregation in foreigners, impairing conformity. Again, despite the model considerations [14], no significant effect on acculturation was detected. Another concern is the difficulty to confront Ha with Hc, isolating mere conformity from social learning due to preferred demonstrators. For example, Kalmijn [35] documents a tendency for children of native-foreigner couples to be more acculturated than those of foreign couples, pointing to higher chances of establishing social networks within the receiving society (preferred demonstrators effect) and residing in less segregated neighbourhoods (conformist effect) as the causes. Table 10 presents an overview of the interpretation of these results.

**Table 10:** Contextualization of the results concerning hypothesis A.

Favourable results	Having contact with a society endorsing the target culture seems to matter, accounting for around 6–8% of the variation in acculturation scores.
Contrary results	However, direct contact with a specific cultural environment does not seem to be determinant for acculturation to occur–as natives who never had contact with their companion’s culture still scored above the scale’s midpoint. Furthermore, the significant difference between natives and foreigners apparently was rather due to a higher relative quantity of friends from the target culture. On the other hand, the relative quantity of friends from the heritage culture did not seem to impair acculturation–contrarily to the relative quantity of friends from third other cultures.
Practical implications	Preventing migrants from isolating from the local society is a first step, but promoting the establishment of a network of close friends within the native culture may be even more efficient in enhancing acculturation. Maintaining close bounds with peers sharing the heritage culture, by itself, does not compromise the acculturation process by any means.
Future research	The differences between contact-no contact groups should be tested using an acculturation scale based on knowledge and participation across several cultural traits. Also, future studies expanding the sample size can help verifying the present results relating pair assortation and time of contact with acculturation.

Detecting a positive effect which connects acculturation and payoff-biased social learning was paramount to sustain Hb, but significance was not attained. Still, it must be taken into account that the proposed measurement has to be improved (S4 Appendix). In fact, the positive association between PRQ’s satisfaction component and acculturation constitutes an unexpected piece of evidence sustaining that payoff-biases may catalyse the acquisition of a new culture. The idea of normative negotiation, inspired in Bunce and McElreath’s model [15], did not seem to apply to the present sample. Only a marginally significant link was detected between normative assortation and cultural maintenance. It may be that models assuming the interaction of populations from different cultural backgrounds are driven by the motivation to avoid peer punishment and maximizing cooperation, are not a suitable to analyse acculturation, at least in this context [36]. Future studies focusing on this aspect might provide clearer answers.

The other aspect assumed to affect behaviour in normative negotiation is cultural conservatism [16], having CTM as a proxy. Whereas the desire component monopolized the significant share of variance with cultural maintenance, CTM-emotion gained relevance regarding acculturation in the foreigner and full samples. Nevertheless, it failed to significantly predict language proficiency (S7 Table), and the medium effect size connecting CTM-desire and acculturation lost its significance when controlling for cultural maintenance. Still, CTM-emotion partially mediated the relation between cultural maintenance and acculturation, which suggests that underlying this component might be a general appreciation for culture and openness to multicultural environments.

Another possibility, not necessarily incompatible, emerged during an interview, when one of the participants protested to the other, while answering the CTM questions: “Well, you can peacefully give low scores in those questions, because your culture is not endangered; with me it is very different. . .” In the present sample the CTM showed variations due the number of children. Thus, contrary to previous indications [17, 27], besides depending perhaps on the education and genetic factors, it seems to be to some extent a latent characteristic, particularly at the emotional level. The feeling of risking cultural extinction, might change some behavioural aspects of the individual. Indeed, Wohl *et al*. [37] observed that the perceived physical or symbolic threats to one’s group enhanced, through collective angst, the desire to engage into ingroup-strengthening behaviours. Accordingly, another study reported a population of second-generation emigrants living abroad who supposedly identified more with the culture of origin than the parents [33]. Even more so, there seems to exist a link between personal and cultural continuity, found in the analysis of demographic data regarding suicide [38]. The present data points that attachment to the basilar cultural identity may influence the acculturation process, namely through emotions. Seemingly, two cultural identities can co-exist without impairing each other by any means. One poor decision taken within the context of Hb, was not to leave the door open for natives to respond to items regarding normative assortation and payoff-biased social learning, providing they have had enough contact with the companion’s cultural background. The discussion of aspects related with Hb is represented in Table 11.

**Table 11:** Contextualization of the results concerning hypothesis B.

Favourable results	Unexpectedly, the satisfaction component of PRQ was positively correlated with acculturation, rather than trust. However, this was only verified in the native sample, suggestion that natives may rely more in payoff-biases, adapting to the partner’s culture in order to improve the quality of life and the cooperation in the household. This may happen through a general mechanism documented to occur in couples, generally, which is the inclusion of the other in the self. Still, it seemingly can work as a vehicle of cultural transmission, payoff-driven. Additionally, both CTM components were positively correlated with acculturation, which in some way suggests that solidifying a cultural identity within a novel society might pay in terms of personal well-being.
Contrary results	No significant relation was obtained between payoff-biased social learning and acculturation, just as normative assortation did not seem to affect the process. Therefore, no direct connection was found in the foreigner sample pointing that acculturation is enhanced by the disposition to seek for beneficial traits.
Practical implications	If the level of acculturation of natives is indeed affected by payoff-biases, then probably it is possible to increase a society’s openness towards foreigners by advocating for the beneficial aspects of living in a multicultural environment.
Future research	The scale concerning payoff-biased social learning needs to be improved, and formulated in such a way that can be applied to natives as well. The normative assortation scale must be redesigned.

Hc received support across several predictions. As expected, an association between PQR and acculturation was verified, though only in the native and full samples. Satisfaction (large-medium effect) and commitment (medium effect) were the salient components. Contrary to expectations, trust was not significant. Nonetheless, these validated ones are aspects plausible to exist in a model-based bias–and they were the very same predictors of a phenomenon common to marital relations, which is the inclusion of the other on the self [39]. In fact, this can be a plausible mechanism acting within model-biased or payoff-biased social learning. There can be a self-feeding loop occurring, specific to this population: commitment enhances acculturation, achieving cultural similarity and understanding in the couple increases satisfaction, which will stimulate the willingness for commitment; and so forth. In fact, one participant stated he made a spontaneous effort to establish traditions from the foreign companion’s culture at home so she would feel better. Being mostly relevant for natives, it can be advanced that PRQ’s potential for cultural transmission may be flexible, increasing when other cues are absent. In one couple it seemed to be one of the major drivers of acculturation: initially, they barely understood each other, hence he was taking Italian lessons with doubled effort and studying with her dedicated help, so they could get to comprehend mutually beyond the simple, unique communication system they had passionately built. Thus, while some affirmed a more independent process, others could humorously blame the partner for getting acculturated; yet building deep social bounds with individuals from a certain culture, not just merely interacting with them, does seem to play a role, potentially explaining 9% of the acculturation scores’ variance in natives. Future studies should check whether this effect extends to close friends. Interestingly, PRQ and the relative quantity of close friends from the companion’s culture explained a similar share of the acculturation scores variance in the full sample, of around 6%. The relative quantity of close friends from the companion’s culture approached marginal significance for natives and foreigners, suggesting that the null hypothesis might be rejected with a larger sample. Although it should be considered cautiously, this association cancelled the marginal one established between the number of years foreigners spent in Italy/Portugal and acculturation, as well as their acculturative advantage towards natives. Again, this illustrates how complicated it is to distinguish conformity from model-biased learning. Another outcome which revealed the importance of preferred demonstrators, was the small effect of the association between the time spent with the companion and acculturation. However, it has emerged exclusively in the full sample, and the extent of the effect was no bigger than that of time of contact obtained in the foreigner sample. Future studies with larger samples can help to better understand these comparisons. The predicted correlation between having strong affective ties with the own family and cultural maintenance follows previous results [6, 40]. Despite it being intuitive that through the family a bridge of contact with the culture of origin can be maintained, enhancing cultural maintenance, this connection was not verified in the foreigner sample. Again, future studies can verify if these effects are indeed asymmetrical. Then, it was pointed out that the quality of the relationship with one’s own family not only predicts intermarriage [21], but it also might play a role in the acculturation process. This is not an obvious link. Nonetheless, it becomes clearer considering that perceived family dysfunction is associated with higher levels of acculturative stress [41]; and that familial lack of cultural coordination is usually a consequence of cultural maintenance gaps, rather than acculturation differentials [42]. The fact that the connection was stronger in foreigners, who should experience acculturative stress more severely, supports this explanation. Similarly, the positive relation between CTM-emotion and acculturation shows the influence of psychological dispositions embedded in an individual’s cultural identity. Still, it must be mentioned that the evidence supporting Hc may be inflated by the weight that the acculturation scale puts on sociability-related factors (S1 Text). Table 12 synthesizes the discussion around Hc.

**Table 12:** Contextualization of the results concerning hypothesis C.

Favourable results	The relative quantity of friends from the target culture accounted close to 7% of the variance in acculturation scores, though exclusively in the full sample. Similar results were obtained for PRQ, in the native (9%) and full (6%) samples. Being satisfaction and commitment the most salient components, this relation falls into a grey zone between payoff biases and model-learning. The payoff-biased hypothesis is favoured, because time spent with the companion, only significant in the full sample, had a smaller effect size. The relationship with one’s own family might help to ameliorate acculturative stress, thus indirectly facilitating cultural learning–which is in line with the fact that it was more relevant with foreigners, who should face more intense levels of acculturative stress. Results concerning CTM-emotion showed that foreigners’ acculturative behaviour is more affected by emotional aspects.
Contrary results	Trust was not one of the salient PRQ’s components, and that would be expected to define a preferred demonstrator. Emotional aspects clearly played a role in the acculturation process, however it may be that they relate as much with payoff biases as with model-learning.
Practical implications	Maintaining close relationships with the family can actually facilitate acculturation, just as long as the individual has contact with demonstrators from the target culture. So, it should be encouraged. Furthermore, by helping foreigners to establish friendships networks within the local society may be a successful tool to boost their cultural adaptation. Finally, be it underlined by payoff biases or model-learning, studying the predictors of acculturative stress and offer support for foreigners to overcome it, seems to be an efficient way to promote cultural transmission.
Future research	Future studies could expand the current sample size to verify the results that were only significant in the full sample. Furthermore, more detailed efforts to untangle payoff biases from model-learning within the context of acculturation could be extremely useful.

Looking at the big picture, the outcomes suggest that contact (whether operating through conformity or random learning) and developing deep bonds with individuals from the culture being acquired (which may itself produce payoff-biases) were mechanisms of cultural transmission, possibly influenced by the ties maintained with one’s heritage culture. Thus, traces of several different social learning mechanisms were detected. In addition to the significant difference between the acculturation scores of natives and foreigners, they diverged on the patterns relating CTM components with acculturation, on the predictive power of PRQ, and on the impact of the affective ties with one’s own family. The fact that language proficiency had some discrepant results in relation to those obtained using the acculturation scale, also supports Hd–because not only does it favour that acculturation recruits several social learning mechanisms, as it suggests the process may change depending on the cultural trait. Additionally, a significant male-female distinction on the average acculturation scores further denotes behavioural flexibility in the process of acquiring a new culture. The present data only detected differences particular to CTM-emotion. According to current literature, hominoid species have the tendency to favour female dispersion, a particularity known as the “ape case”, as this sex may gain advantage in inter-generational reproductive conflicts by migrating [43, 44]. Having psychological mechanisms predisposing them to be more permeable to acquire another culture, could explain the females’ acculturative advantage, and why in a previous study women had a greater capacity to embrace the collectivistic nature of a different society [8]. In fact, migrant women seem to be more efficient in marrying natives, because Italian men are almost three times more likely to engage in inter-marriage than the opposite sex, across regions [23]. Finally, previous acculturation studies had detected a greater tendency for males to exhibit behavioural problems, whereas females were merely more vulnerable to psychological distress [6]. Table 13 frames these results in a simplified manner.

**Table 13:** Contextualization of the results concerning hypothesis D.

Favourable results	Different results were obtained between the native and the foreigner samples; and between sexes. In addition, acculturation patterns may change according to the cultural trait considered. This demonstrates a great behavioural flexibility and diversity concerning the adaptation to a new culture, as predicted. Traces of payoff-biases, model-learning, and even conformity were found.
Contrary results	Some results were unclear, especially when significant only in the full sample.
Practical implications	Integration programs might benefit from designing some sex-specific strategies. Furthermore, they can potentially be more efficient if they access which cultural traits does the local population is more sensitive about–just as they should investigate which cultural traits bring more well-being for migrants to adapt to. Some cultural traits might have unpredictably positive high impact on acculturation–for instances, learning the dances of the culture can help building a strong network of friendships within it, and further indirectly enhance acculturation through model-learning.
Future research	Further examinations are necessary to produce a clearer picture of which cultural transmission mechanisms can be distinguished, their range of influence, and the context in which they become salient. Finally, future studies should determine which cultural traits should be prioritized in order to facilitate acculturation to occur–for example, by comprehending the local language, all the other cultural aspects become easier to be learned.

After testing all the hypotheses, it was statistically shown that pair and normative assortation were not exclusively dictated by CTM. The active desire to preserve the culture of origin related more to normative preferences, whilst the emotional sphere was particularly associated with the inclination to interact with individuals from the same culture. Pair assortation is also dictated by external factors, such as the availability of pairs from the same culture and the native’s willingness to interact with foreigners. In turn, normative assortation may depend on the second culture’s characteristics and collisions of certain local norms with personal values and social tastes, independently of the disposition to maintain and transmit the culture of origin. Interestingly, the native’s relative quantity of friends from third other cultures was negatively associated with acculturation. Perhaps it disperses the acculturative focus, or multicultural environments may reduce the will to identify with any culture in particular. Nonetheless, it is clear that the general willingness to interact with people from other cultures is not necessarily associated with high levels of acculturation to a given culture. Lopez-Class *et al*. [25] proposed a list of external factors potentially affecting acculturation: the existence of ethnic enclaves and the dominant culture’s values, behaviour, and attitudes, which may change according to the native’s perception of the motive for migration [45]. In fact, several of the present results point to the importance of assessing the behaviour of the receiving society towards foreign cultures; which exerts great influence on the cultural orientation of foreigners [46]. Hence, it is suggested that Carlson and Güler’s [34] conception of *cultural preference* could be used to place the local society within a continuum, from allowing the substitution of the majority’s culture, to demanding foreigners for cultural assimilation. Nevertheless, the measurement would have to suffer modifications, not be so identical to the present acculturation and cultural maintenance items. Here, items from the CTM-desire could be borrowed, and cultural preference take its place. On the other hand, Ward and Szabó [6] suggested that the internal factors which influence acculturation are personality traits, the intercultural “mindsets” (the capacity to distinguish and understand cultural differences), and cultural intelligence (the ability to efficiently adapt to new cultural environments). As previously mentioned, here, language proficiency was excluded, as it is an inherent part of the acculturation process.

This study has some limitations. First, it was not possible to attain the desired power for all the analyses. This may be why some smaller effects were only detected in the full sample. Also, there is a chance that some false positive was obtained. However, the amount of tests performed in each sample does not justify the use of multiple-test correction methods. Second, participants might have taken the present acculturation scale essentially as a measure of the general openness to interact with people from their own and their companion’s cultures. Individuals that never had direct contact with their companion’s heritage culture might have substantial scores just for being willing to work, be friends, and participate in social activities with the companion and countrymen alike. It is not that the measure lacks validity, it just discards, to some extent, several important cultural aspects, such as knowledge about institutions, traditions, and artistic aspects. A more fine-grained approach for cultural learning studies could be based on knowledge and actual participation in a set of cultural traits taken from Human universals’ list–the cultural practices and institutions shared by virtually all known societies [47]. Third, the PRQ scale could also have been better adapted to this framework, and used together with other scales of dominance and prestige, developed by Cheng *et al*. [48] to define the status of an influential demonstrator [49]. Fourth, the acculturation domain (public or private) and cultural distance (the degree of dissimilarity between the Italian/Portuguese and the foreign cultures) could also have been accounted for [46], despite no significant differences being observed between the Italian and Portuguese samples.

## Conclusion

Evolutionary researchers frequently depict cultural transmissions as an osmotic-like process leading migrants to adopt the rule of the majority. However, the present results suggest that the contact effect is as relevant as creating affective ties with models to learn from. Hence, evolutionary models should consider other forces driving acculturation, together with conformity. In fact, the native’s beliefs and attitudes towards immigrants should be assessed as a key point to establish if the receiving society demands for assimilation or tolerates integrative orientations, which potentially constitutes two distinct scenarios. According to this study, factors related with the negotiation of norms, such as normative assortation and payoff-biased social learning, need further empirical examination before they can be efficiently included in acculturation models. Nevertheless, the PRQ, namely the satisfaction and commitment components, seem to have the potential to stimulate acculturation, especially when other cues are absent. The salience of satisfaction unexpectedly supported that payoff-biases might have played a part in the obtained outcomes. Then, rather than pair assortation or the general willingness to interact with people from other cultures, it was the battery of close friends specifically from the culture being acquired that seemed to enhance cultural transmission. This highlights the importance of the natives’ willingness to interact with foreigners, acting as preferred demonstrators. As a consequence, prejudice and segregation should actually fuel cultural shocks. Additionally, support from one’s own family, probably by reducing the acculturative stress, seem to facilitate acculturation. This piece of evidence, together with the positive effect of CTM-emotion, re-affirms the relevance of the affective dimension embedded within the cultural identity. Finally, it is suggested that evolutionary scientists can benefit from adapting methods from cross-cultural psychology and sociology to verify the external validity of culture transmission models, and further investigating the emotional dimension of social learning.

## Supporting information

S1 Appendix Measurements’ validation. (PDF)

S1 Dataset The full dataset used in the study. (DTA)

S1 Fig Outliers within the main variables from the native sample. The circles represent the outliers, whereas the star marks the far-out outliers. In both cases, the number identifies a specific data point.
(TIF)

S2 Fig Outliers within the main variables from the foreigner sample. The circles represent the outliers, whereas the star marks the far-out outliers. In both cases, the number identifies a specific data point.
(TIF)

S3 Fig Scatter plots with fit line for the acculturation predictors that were significant or marginally significant in the foreigner sample. (TIF)

S4 Fig Scatter plots with fit line for the acculturation predictors that were significant or marginally significant in the native sample. (TIF)

S1 Table Measurement invariance verification for the main constructs. (PDF)

S2 Table Excluded cases’ report. (PDF)

S3 Table Report of the deviant cases considered. (PDF)

S4 Table Normal distributions detected in the foreigner sample. (PDF)

S5 Table Associations between cultural maintenance and the main factors considered. (PDF)

S6 Table Variance explained regarding cultural maintenance. Obtained through multiple regressions, using the relevant factors within each sample.
(PDF)

S7 Table Associations between language proficiency and the main factors considered. (PDF)

S8 Table Variance explained regarding language proficiency. Obtained through multiple regressions, using the relevant factors within each sample.
(PDF)

S9 Table Variance explained regarding acculturation. Obtained through multiple regressions, using the relevant factors within each sample.
(PDF)

S1 Text The questionnaire in English. (PDF)

S2 Text The Portuguese version of the questionnaire. (PDF)

S3 Text The Italian version of the questionnaire. (PDF)
