# Supplementary material for: Examining acculturation in mixed-couples to test cultural transmission mechanisms
Source: PLoS One. 2022 Apr 6;17(4):e0266229. doi: 10.1371/journal.pone.0266229 (PMC8985958; doi:10.1371/journal.pone.0266229)
Supplement: S1 Appendix — (PDF) [file pone.0266229.s001.pdf]

S1 Appendix. Measurements' validation.

A. Normative assortation

Initial Cronbach's α = .56

After deleting the questions "I frequently feel troubled understanding some of the Italian/Portuguese ways of interaction", Cronbach's α = .59

KMO = .60 (data is in the border miserable-mediocre for factor analysis)

Marker item: "I would rather behave as I was taught within my heritage culture than in the Italian way" - because of the low reliability and homogeneity, only this item was retained for the analyses concerning this construct.

B. Payoff-biased social learning

Cronbach's α = ,77.

KMO = ,67 (mediocre data for factor analysis).

Total item response variance explained: 47.09%.

Nonredundant residuals superior to .05: 66% (problematic).

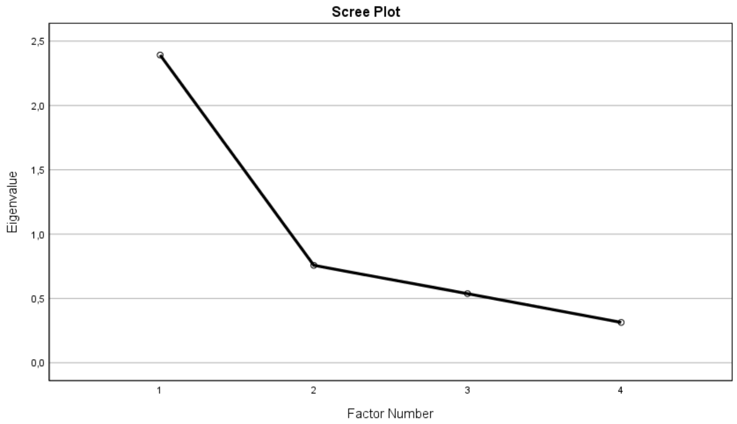

Scree plot for the payoff-biased social learning.

The scree plot points to a single-factor model.

Marker item: “Actions that in my heritage culture would be considered normal were often judged with disapproval”, arguably the item that better captures the concept being measured.

C. Culture-transmission motive (validated on full sample, others had similar results)

Initial Cronbach’s α = .82

After deleting the question “My family would prefer that I married someone from my culture of origin”, Cronbach’s α = .85

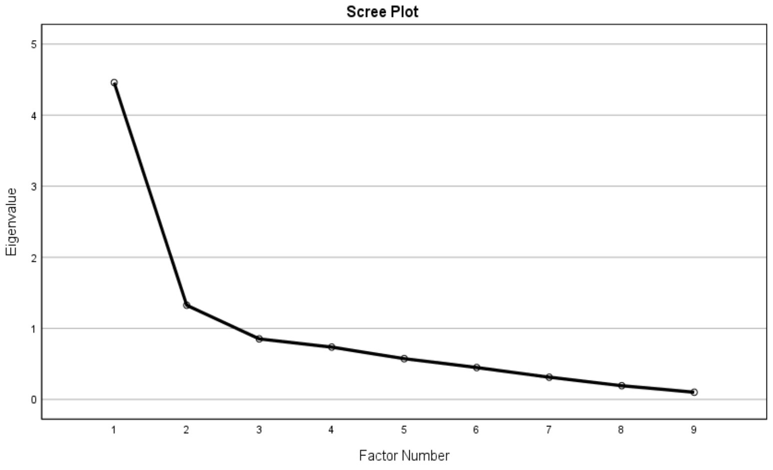

Scree plot for the culture-transmission motive.

Initial KMO = .81 (data is meritorious for factor analysis)

Initial nonredundant residuals superior to .05: 66%

Scree-plot points for the existence of two factor underlying the data. The factor plot later corroborated this.

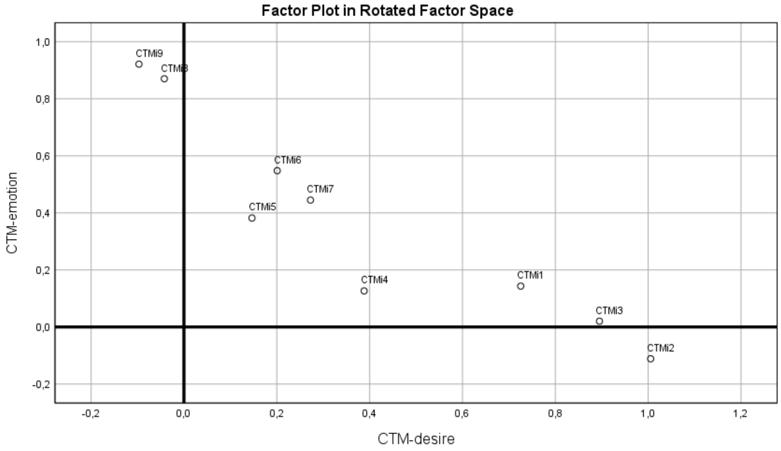

Factor plot for the components desire (factor 1) and emotion (factor 2).

Thus, it was decided to remove the item “If a countryman acquaintance is in trouble, I should provide help”, which was the only one referring to CTM-action and run the exploratory factor analysis again, now extracting two factors (CTM-desire, CTM-emotion) using promax rotation with kappa = 4.

Final KMO = .80

Final item response variance explained: 45.56% + 11.84% (57.40% total)

Final nonredundant residuals superior to .05: 8%

There were no problematic cross-loadings, assuming the .32 threshold. The item “Would you feel sorry if your (present or future) children would forget or lose the language your parents spoke?” loaded on CTM-desire instead of CTM-emotion, therefore having been taken as the desire to transmit the language to the children, rather than the feelings that would come for their rejection of this cultural aspect.

Desire marker item: “Do you wish your culture of origin to be kept alive in the generation of your (present or future) children?”

Emotion marker item: “How do you feel when something positive is reported on the television, radio, or the newspapers about your family’s country or culture of origin?”

Factors correlation: r = .52

D. Perceived Relationship Quality (validation done on the full sample, other had similar results)

An initial analysis run on AMOS pointed that the errors of items measuring a component should not be treated as independent. Thus, the scores were calculated using AMOS.

$\chi^2 = 161.52$; df = 75 ; p < .001 (not favourable)

CFI = .96 (good model fit)

RMSEA = .09, 90% confidence interval $\in$ [.07, .11] (lower bound met acceptable fit)

E. Cultural maintenance (validated on the full sample, other samples had similar results)

Cronbach’s α = .90

KMO = .89 (meritorious data for factor analysis)

Total item response variance explained: 50.79%

Nonredundant residuals superior to .05: 50%

The scree plot supports the single-factor model.

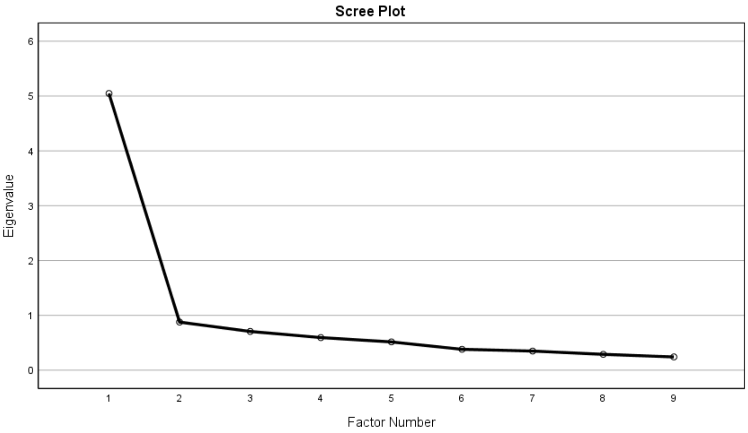

Scree plot for cultural maintenance.

Marker item: “It is important to me to maintain or develop the practices of my heritage culture”, which is positive because it indicates that the scale is not putting excessive weight in items related with sociability.

F. Acculturation (validation done on the full sample, other samples had similar results)

Cronbach’s α = .89

KMO = .88 (meritorious data for factor analysis)

Total item response variance explained: 46.54%

Nonredundant residuals superior to .05: 41%

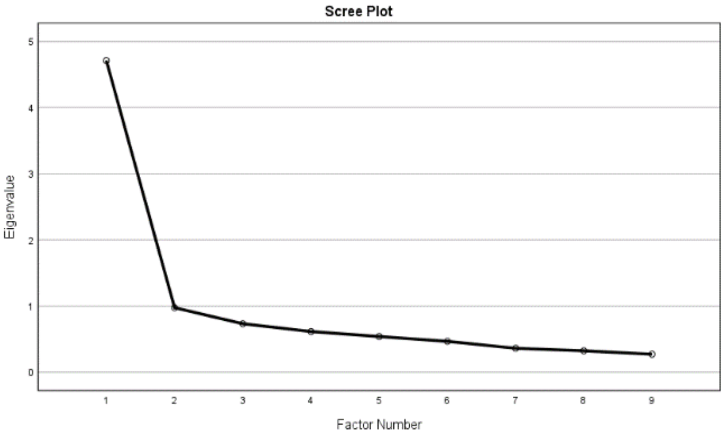

Scree plot for acculturation.

The scree plot supports the single-factor model.

Marker item: “It is important to me to maintain or develop the practices of my companion's culture”, which is positive because it indicates that the scale is not putting excessive weight in items related with sociability.
