# Supplementary figures and images for: Examining acculturation in mixed-couples to test cultural transmission mechanisms
Source: PLoS One. 2022 Apr 6;17(4):e0266229. doi: 10.1371/journal.pone.0266229 (PMC8985958; doi:10.1371/journal.pone.0266229)

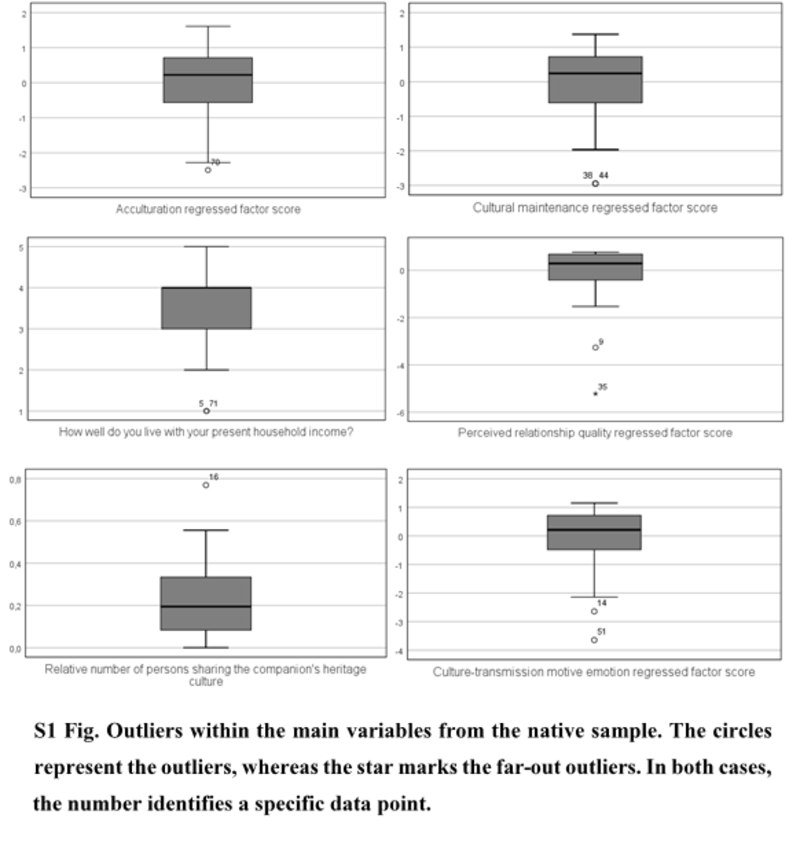

Supplement: S1 Fig — The circles represent the outliers, whereas the star marks the far-out outliers. In both cases, the number identifies a specific data point. (TIF) [file pone.0266229.s003.tif]

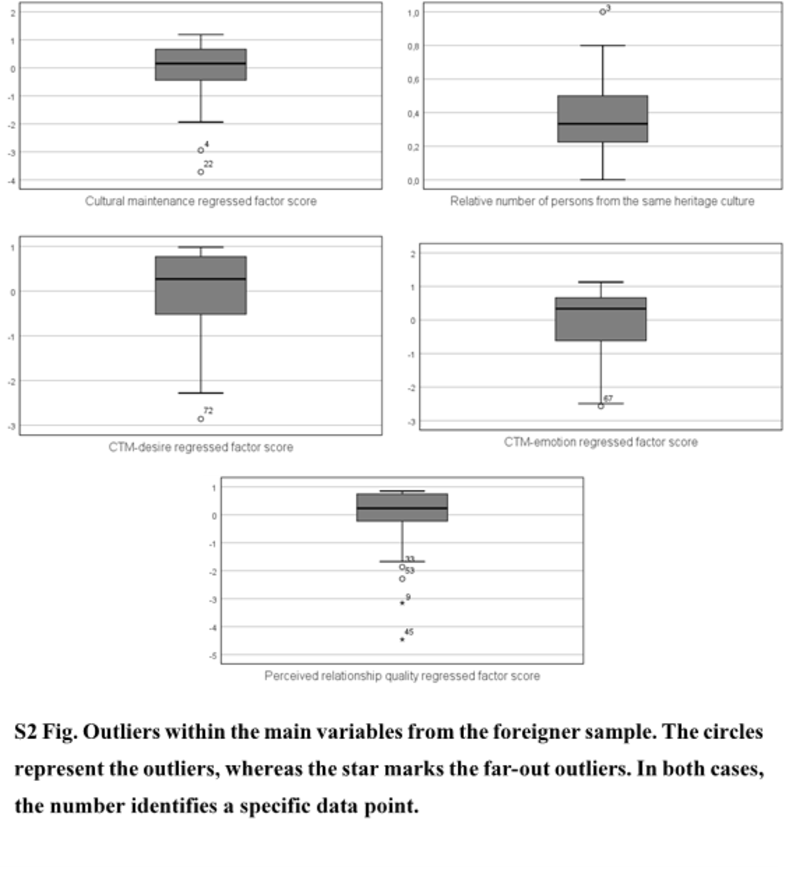

Supplement: S2 Fig — The circles represent the outliers, whereas the star marks the far-out outliers. In both cases, the number identifies a specific data point. (TIF) [file pone.0266229.s004.tif]

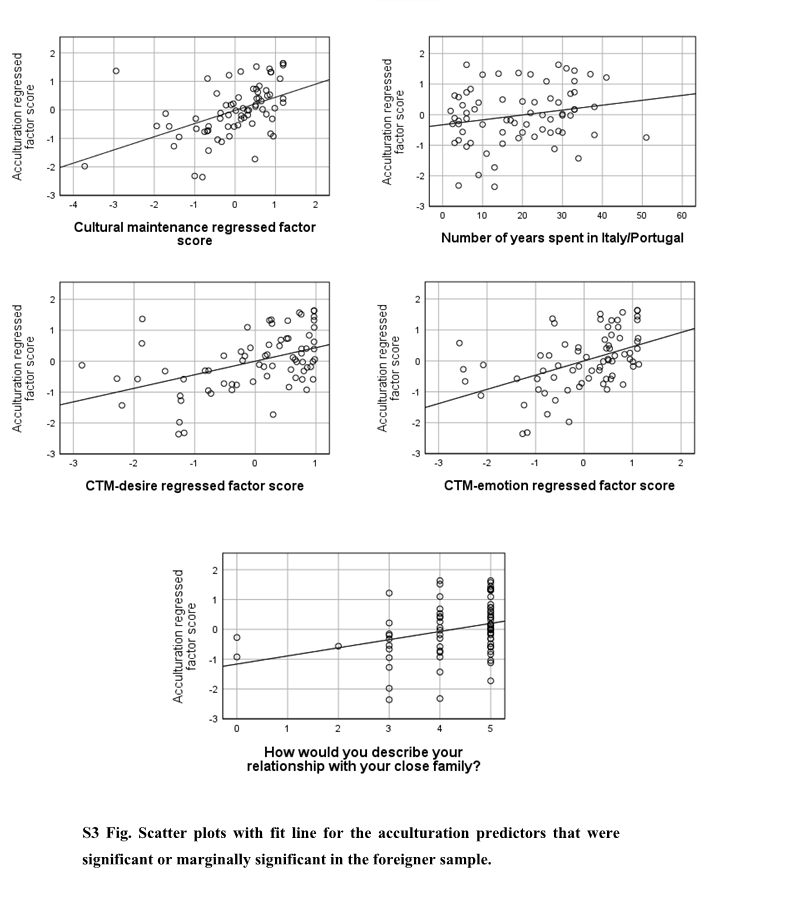

Supplement: S3 Fig — (TIF) [file pone.0266229.s005.tif]

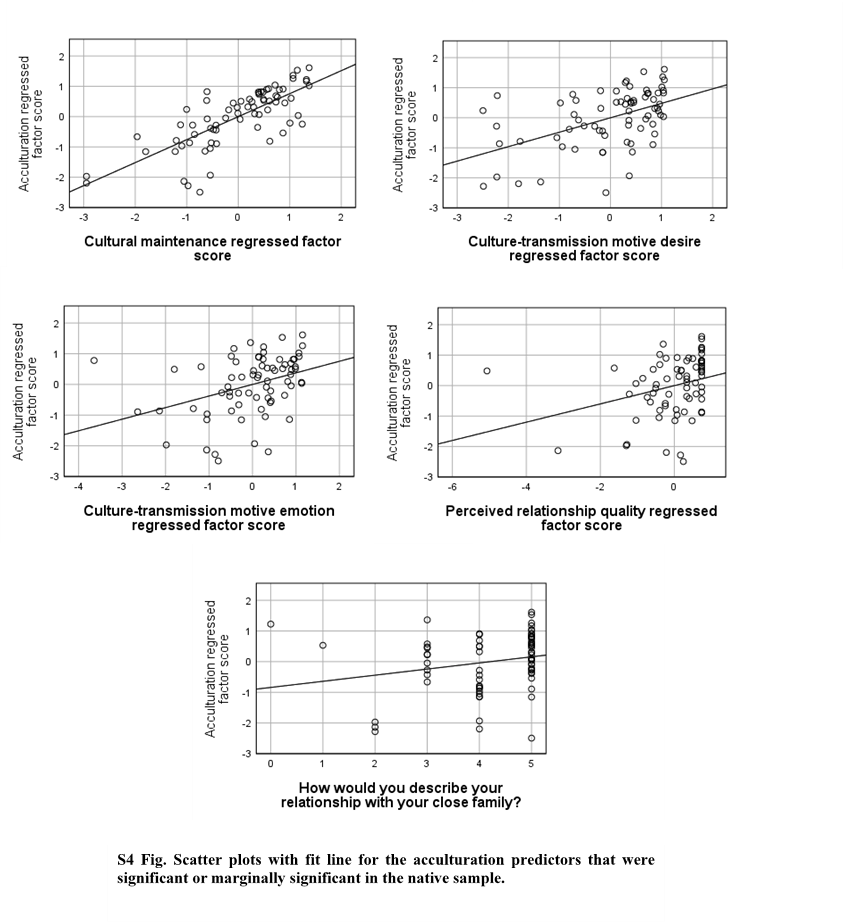

Supplement: S4 Fig — (TIF) [file pone.0266229.s006.tif]
