# Supplementary material for: Examining acculturation in mixed-couples to test cultural transmission mechanisms
Source: PLoS One. 2022 Apr 6;17(4):e0266229. doi: 10.1371/journal.pone.0266229 (PMC8985958; doi:10.1371/journal.pone.0266229)
Supplement: S1 Table — (PDF) [file pone.0266229.s007.pdf]

S1 Table. Measurement invariance verification for the main constructs.

		χ^2 (df)	CFI	RMSEA, with 90% confidence interval	Invariance attained?
Acculturation	M1	116.22 (54) highly significant	.89 dubious fit	.09 ∈ [.07, .11] lower bound reaches acceptable fit	Yes
	M2	Δ=7.19 (8) non-significant	.89 dubious fit	.08 ∈ [.06, .11] acceptable fit	Yes
	M3	Δ=6.18 (8) non-significant	.89 dubious fit	.08 ∈ [.06, .10] acceptable fit	Yes
Payoff-biased social learning	M1	8.60 (4) non-significant	.94 acceptable fit	.13 ∈ [.00, .25] lower bound reaches good fit	Yes
	M2	Δ=5.10 (3) non-significant	.91 acceptable fit	.12 ∈ [.00, .21] lower bound reaches good fit	Yes
	M3	Δ=4.37 (3) non-significant	.90 acceptable fit	.11 ∈ [.00, .12] lower bound reaches good fit	Yes
Perceived relationship quality	M1	288.64 (150) highly significant	.94 acceptable fit	.08 ∈ [.07, .10] acceptable fit	Yes
	M2	Δ=34.12 (14) significant	.93 acceptable fit	.08 ∈ [.07, .10] acceptable fit	Yes*
	M3	Δ=20.03 (14) non-significant	.93 acceptable fit	.08 ∈ [.07, .09] acceptable fit	Yes
CTM-desire	M1	8.30 (4) marginally significant	.99 good fit	.09 ∈ [.00, .17] lower bound reaches good fit	Yes
	M2	Δ=2.50 (3) non-significant	.99 good fit	.06 ∈ [.00, .13] lower bound reaches good fit	Yes
	M3	Δ=2.52 (3) non-significant	.99 good fit	.05 ∈ [.00, .17] good fit	Yes
CTM-emotion	M1	46.70 (18) highly significant	.90 acceptable fit	.11∈ [.07, .14] lower bound reaches acceptable fit	Yes
	M2	Δ=7.99 (5) non-significant	.89 dubious fit	.10 ∈ [.07, .13] lower bound reaches acceptable fit	Yes
	M3	Δ=4.10 (5) non-significant	.90 acceptable fit	.09 ∈ [.06, .12] acceptable fit	Yes

M1: Configurational invariance; M2: Metric invariance; M3: Scalar invariance. *The $\Delta\chi^2$ obtained was significant, which is problematic; however, as the condition ΔCFI < .01 was verified and the RMSEA value was maintained, it is reasonable to proceed with the invariance analyses.
