# Supplementary material for: Examining acculturation in mixed-couples to test cultural transmission mechanisms
Source: PLoS One. 2022 Apr 6;17(4):e0266229. doi: 10.1371/journal.pone.0266229 (PMC8985958; doi:10.1371/journal.pone.0266229)
Supplement: S2 Table — (PDF) [file pone.0266229.s008.pdf]

S2 Table. Excluded cases' report.

**Participant(s)**	**Reason for being excluded**
ROMFN11	Met the partner abroad, and they spent most of the time together outside Italy.
MILFY19+MILMN19	They met in Italy, but spend the time together between Italy and a third country. Furthermore, it is a quite recent relationship, lived at distance to a great extension.
TORMN20	Left most of the time together with the spouse in his home country, only very recently has he experimented to be the "foreigner."
On3MY	Other than being divorced, stated his heritage culture as being the foreign one, despite being native. Having provided no clarifications, should be excluded.
On5FY + On6FN	Failed to provide consent.
On22M	He is Italian and met his foreign companion in Portugal, so they are both foreigners living in a third country.
On36M	It is completely unclear which is the heritage culture and the culture of origin of this participant.
ptOn4FNcB + ptOn6MYcB	The Portuguese of the couple was born in France and only moved to Portugal with 23 years.
ptOn5MY	Not a mixed couple.
ptOn17MN	Relevant information such as length of stay in Portugal and the culture of origin are missing.
ptOn18F	Born in France, raised by Portuguese parents (under their culture of origin). Came to Portugal only on her twenties. No clear role native-foreigner.
ptOn37FN	Daughter of a Portuguese-French couple. The Portuguese influence during childhood was not predominant but strong. No clear role native-foreigner.
ptOn39MNcR + ptOn56FYcR	This couple lived most of their relationship at distance or in a third country.
ptOn53F	Heritage and culture of origin are not clear.
ptOn59FN	Daughter of a Portuguese-French couple. The Portuguese influence was strong, especially having the family moved to Portugal when she was 2. Not a suitable foreigner.
