# Supplementary material for: Examining acculturation in mixed-couples to test cultural transmission mechanisms
Source: PLoS One. 2022 Apr 6;17(4):e0266229. doi: 10.1371/journal.pone.0266229 (PMC8985958; doi:10.1371/journal.pone.0266229)
Supplement: S3 Table — (PDF) [file pone.0266229.s009.pdf]

**S3 Table. Report of the deviant cases considered.**

| Participant(s) | Reason for still being considered |
|---|---|
| BOLFY3 | Her mother was British, but the participant ensures having been raised exclusively within the Italian culture. |
| ROMFY12 | Met the partner abroad, and spent the time 50-50 between that foreign country and Italy. |
| ALBMY23s | Went very young to the wife's home country, were they met after some years. Ensured his was education strictly Italian, especially because his parents were very conservative. |
| VERFN26 | She was actually born in Italy, but left back to her parents' home country with 2 months. Her family is very culturally-conservative and the education was traditional. |
| On2FN + On19FY + On40FNcG + On41MYcG | These participants got divorced at around 2 years from the date of participation. As their marriages endured in approximately 30 years, it is reasonable to consider them. |
| On24FN | Went living to Italy with 27 years, stayed there for 6 years and then they lived for around 30 years in countries very near Italy, returning very frequently. |
| ptOn27FN | Granddaughter of Portuguese emigrants, but declares both the culture of origin and the heritage one were the French. Furthermore spent half of the life living in France. |
| ptOn30MYcL + ptOn46FNcL | This couple spent almost the same time between Portugal and the foreign country. Just one year less in Portugal. |
