# Supplementary material for: Examining acculturation in mixed-couples to test cultural transmission mechanisms
Source: PLoS One. 2022 Apr 6;17(4):e0266229. doi: 10.1371/journal.pone.0266229 (PMC8985958; doi:10.1371/journal.pone.0266229)
Supplement: S4 Table — (PDF) [file pone.0266229.s010.pdf]

S2 Table: Presentation of the constructs exhibiting a normal distribution. All are exclusive to the foreigner sample.

	Kolmogorov-Smirnov (sig)	Shapiro-Wilk (sig)
Acculturation score (72 df)	.06 (.20)	.98 (.19)
Relative number of friends from the same heritage culture (71 df)	.10 (.07)	.97 (.12)
Relative number of friends from the companion's heritage culture (71 df)	.09 (.20)	.97 (.13)
