# Supplementary material for: Examining acculturation in mixed-couples to test cultural transmission mechanisms
Source: PLoS One. 2022 Apr 6;17(4):e0266229. doi: 10.1371/journal.pone.0266229 (PMC8985958; doi:10.1371/journal.pone.0266229)
Supplement: S5 Table — (PDF) [file pone.0266229.s011.pdf]

S5 Table. Associations between cultural maintenance and the main factors considered.

	Natives	Foreigners	Full sample
Acculturation	r(69) = .75<br>$p < .001$	r(70) = .48<br>$p < .001$	r(141) = .62<br>$p < .001$
Contact (yes/no)	$\eta_p^2 = .001$<br>p = .78	-	-
Time of contact	β = .04<br>p = .75	β = .13<br>p = .30	-
Pair Assortation	β = .17<br>p = .16	β = .21<br>p = .09	β = .15<br>p = .07
Payoff-biased Social Learning	-	β = -.09<br>p =.47	-
Normative Assortation	-	β = .23<br>p = .06	-
CTM-desire	β = .64<br>$p < .001$	β = .76<br>$p < .001$	β = .69<br>$p < .001$
CTM-emotion	β = .39<br>p = .001	β = .46<br>$p < .001$	β = .44<br>$p < .001$
Perceived Relationship Quality	β = .18<br>p = .13	β = -.14<br>p = .26	β = -.01<br>p = .87
Friends from the companion's culture	β = .07<br>p = .56	β = -.26<br>p = .03	β = -.09<br>p = .28
Time spent together	β = .10<br>p = .42	β = .22<br>p = .08	β = .15<br>p = .08
Relationship with the own family	β = .26<br>p = .04	β = .14<br>p = .26	β = .20<br>p = .03
Friends from third other cultures	β = -.26<br>p = .03	β = .11<br>p = .35	β = -.09<br>p = .28

Contact's effect was determined using GLMs, whereas the other analyses relied in linear regressions; all controlling for subjective SEC.
