# Supplementary material for: Examining acculturation in mixed-couples to test cultural transmission mechanisms
Source: PLoS One. 2022 Apr 6;17(4):e0266229. doi: 10.1371/journal.pone.0266229 (PMC8985958; doi:10.1371/journal.pone.0266229)
Supplement: S6 Table — Obtained through multiple regressions, using the relevant factors within each sample. (PDF) [file pone.0266229.s012.pdf]

S6 Table. Variance explained regarding cultural maintenance. Obtained through multiple regressions, using the relevant factors within each sample.

	Factors considered	Variance explained without acculturation	Variance explained including acculturation
Natives	Friends from the third other cultures, both CMT components, Relationship with the family	48%	70%
Foreigners	Time spent together, Friends from the same and the companion's culture, both CTM components, Normative assortation	64%	68%
Full sample	Time spent together, Pair assortation, CMT-desire and emotion, Relationship with the family	53%	64%

Marginally significant factors were considered. Subjective SEC integrated all the regressions as control variable.
