# Supplementary material for: Examining acculturation in mixed-couples to test cultural transmission mechanisms
Source: PLoS One. 2022 Apr 6;17(4):e0266229. doi: 10.1371/journal.pone.0266229 (PMC8985958; doi:10.1371/journal.pone.0266229)
Supplement: S7 Table — (PDF) [file pone.0266229.s013.pdf]

S7 Table. Associations between language proficiency and the main factors considered.

	Natives	Foreigners	Full sample
Cultural maintenance	β = .13<br>p = .23	β = .06<br>p = .57	β = .06<br>p = .45
Contact (yes/no)	η_p^2 = .027<br>p = .19	-	-
Time of contact	β = .36<br>p = .004	β = -.21<br>p = .47	-
Conformism	Median: 15.0 (2.5-70), 12.5% had null score (exclusive to natives)		
Pair assortation	β = -.06<br>p = .57	β = -.13<br>p = .20	β = -.28<br>p < .001
Payoff-biased social learning	-	β = -.05<br>p = .60	-
Normative assortation	-	β = -.03<br>p = .80	-
CTM-desire	β = .07<br>p = .50	β = .05<br>p = .63	β = .08<br>p = .29
CTM-emotion	β = .12<br>p = .30	β = -.03<br>p = .75	β = .04<br>p = .62
Perceived relationship quality	β = .06<br>p = .63	β = -.001<br>p = .99	β = .05<br>p = .52
Time spent together	β = .60<br>p = .004	β = -.05<br>p = .83	β = .59<br>p = .002
Friends from the companion's culture	β = .31<br>p = .006	β = .09<br>p = .39	β = .42<br>p < .001
Relationship with the own family	β = .11<br>p = .31	β = .03<br>p = .77	β = .10<br>p = .20
Friends from other cultures	β = -.17<br>p = .12	β = .05<br>p = .64	β = -.14<br>p = .08
Sex	η_p^2 = .031, p = .04 (females maintained the advantage)		

The effects of contact (yes/no) and sex were obtained using GLMs. The remaining analyses relied in linear regressions. Control variables: age, age of arrival (exclusively for foreigners), years of education, number of children had with the current companion.
