# Supplementary material for: Examining acculturation in mixed-couples to test cultural transmission mechanisms
Source: PLoS One. 2022 Apr 6;17(4):e0266229. doi: 10.1371/journal.pone.0266229 (PMC8985958; doi:10.1371/journal.pone.0266229)
Supplement: S8 Table — Obtained through multiple regressions, using the relevant factors within each sample. (PDF) [file pone.0266229.s014.pdf]

S8 Table. Variance explained regarding language proficiency. Obtained through multiple regressions, using the relevant factors within each sample.

	Factors considered	Variance explained
Natives	Total number of months spent in the companion's country of origin, Time spent with the companion, Relative quantity of friends form the companion's culture	42%
Foreigners	(control variables only)	42%
Full sample	Time spent with the companion, Pair assortation, Friends form the companion's culture, Friends from third different cultures	35%

Marginally significant factors were considered. Control variables: age, age of arrival (exclusively for foreigners), years of education, number of children had with the current companion.
