# Supplementary material for: Examining acculturation in mixed-couples to test cultural transmission mechanisms
Source: PLoS One. 2022 Apr 6;17(4):e0266229. doi: 10.1371/journal.pone.0266229 (PMC8985958; doi:10.1371/journal.pone.0266229)
Supplement: S9 Table — Obtained through multiple regressions, using the relevant factors within each sample. (PDF) [file pone.0266229.s015.pdf]

S9 Table. Variance explained regarding acculturation. Obtained through multiple regressions, using the relevant factors within each sample.

	Factors considered	Variance explained without cultural maintenance	Variance explained including cultural maintenance
Natives	Contact (yes/no), Time spent with the companion, PQR, Friends form the companion's culture, CMT-desire and emotion, Relationship with the family	35%	66%
Foreigners	Years spent in Italy/Portugal, Friends form the companion's culture, CMT-desire and emotion, Relationship with the family	36%	42%
Full sample	Time spent with the companion, PQR, Friends form the companion's culture, CMT-desire and emotion, Relationship with the family	38%	55%

Marginally significant factors were considered. Factors that approached marginal significance and were significant in the full sample also integrated the analyses.
