# Supplementary material for: Examining acculturation in mixed-couples to test cultural transmission mechanisms
Source: PLoS One. 2022 Apr 6;17(4):e0266229. doi: 10.1371/journal.pone.0266229 (PMC8985958; doi:10.1371/journal.pone.0266229)
Supplement: S1 Text — (PDF) [file pone.0266229.s016.pdf]

S1 Text. The English version of the questionnaire.

Section A - In this section some brief demographic questions will be asked:

1. Sex

2. Age

3. Years of education

4. Have you been employed in the last 6 months?

5. With your present household income… (“1=is very difficult to live”, “2=is difficult to live”, “3=we are coping”, “4=we are living comfortably”, “5=we are living very comfortably”)

6. Born in Italy? a. If yes: total number of months spent in your companion’s country of origin or countries with a similar culture

b. If no: i) age of arrival in Italy; ii) number of years spent living in Italy

7. Within the last 10 years, in which Italian city did you spent most time living?

8. For how long have you and your companion been together?

9. Are you married with your current partner?

10. Have you been formerly married with someone from the same country of origin as your companion?

11. How many children have you had?

12. How many children have you had with your actual companion?

13. In a scale from “1=Very bad” to “5=Very good” with “0=I don’t maintain contact with them”, how would you describe your overall relationship with your parents and close family?

14. Regarding your companion’s maternal language (answer both using percentages, %): a. How much of it can you understand when spoken?

b. How much can you produce when you want to express through it?

15. Please state which culture(s) do you feel belonging to (or write “none” if that is the case).

Section B - Many of these questions will referrer to your “heritage culture”, meaning the culture that influenced you most (other than your companion’s culture). It may be the culture of your birth, the culture in which you have been raised, or another culture that forms part of your background. If there are several of these cultures, please pick the one that has influenced you most (e.g. Irish, Chinese, Mexican). If you do not feel that you have been influenced by any other culture than your companions’ one, please try to identify a culture that may have had an impact on previous generations of your family. Please answer the following questions in a scale from “1=Strongly disagree” to “9=Strongly agree”:

1. I often participate in my heritage cultural traditions

2. I often participate in my companion’s cultural traditions

3. I enjoy social activities with people from the same heritage culture as myself

4. I enjoy social activities with people from the same heritage culture as my companion

5. I am comfortable working with people of the same heritage culture as myself

6. I am comfortable working with people of the same heritage culture as my companion

7. I enjoy entertainment (movies, music, etc…) from my heritage culture

8. I enjoy entertainment (movies, music, etc…) from my companion’s culture

9. I often behave in ways that are typical to my heritage culture

10. I often behave in ways that are typical to my companion’s culture

11. It is important to me to maintain or develop the practices of my heritage culture

12. It is important to me to maintain or develop practices of my companion’s culture

13. I believe in the values of my heritage culture

14. I believe in the mainstream values from my companion’s culture

15. I enjoy the jokes and humour of my heritage culture

16. I enjoy typical jokes and humour from my companion’s culture

17. I am interested in having friends from my heritage culture

18. I am interested in having friends from my companion’s culture

- Please specify, on the answer sheet under section B, which did you consider to be your heritage culture

Section C - The following items refer to your perceptions of the relationship you have with your companion. Answer in a scale from “1=not at all” to “7=extremely”:

1. How satisfied are you with your relationship?

2. How content are you with your relationship?

3. How happy are you with your relationship?

4. How committed are you to your relationship?

5. How dedicated are you to your relationship?

6. How devoted are you to your relationship?

7. How intimate is your relationship?

8. How close is your relationship?

9. How connected are you to your partner?

10. How much do you trust your partner?

11. How much can you count on your partner?

12. How dependable is your partner?

13. How much do you love your partner?

14. How much do you adore your partner?

15. How much do you cherish your partner?

Section D - This section refers to attitudes towards your culture of origin – the predominant culture endorsed by your parents. So, “family” here refers to them and other close kin. People sharing this culture but that are not kin will be referred as “countrymen”:

1. How important to you are the values and norms of your culture of origin —its ideas about the right way to live, its beliefs about what is proper and what not? (“0=not important at all”, “7=very important”)

2. Do you wish your culture of origin to be kept alive in the generation of your (present or future) children? (“0=I do not care at all”, “7=I wish this very much”)

3. Would it be important for you to maintain your family’s culture of origin and pass it on to your children? (“0=not important at all”, “7=very important”)

4. Would you feel sorry if your (present or future) children would forget or lose the language your parents spoke? (“0=I would not mind”, “7=I would feel very sorry”)

5. Would you feel sorry if your (present or future) children would turn their back on the religion of your family/the non-religious worldview of your family? (“0=I would not mind”, “7=I would feel very sorry”)

6. How much would you regret the loss of norms and values of your family? (“0=I would not mind”, “7=I would feel very sorry”)

7. A hypothetical question: Would you feel sorry if—for whatever reason—your culture of origin would not exist anymore 500 years from now? (“0=I would not mind”, “7=I would feel very sorry”)

8. How do you feel when something negative is reported on the television, radio or the newspapers about your family’s country or culture of origin? (“0=I don’t mind at all”, “7=this makes me very angry”)

9. How do you feel when something positive is reported on the television, radio, or the newspapers about your family’s country or culture of origin? (“0=I don’t mind at all”, “7=this makes me very happy”)

10. My family would prefer that I married someone from my culture of origin (“0=do not agree at all, 7=agree completely”)

11. If a countryman acquaintance is in trouble, I should provide help (“0=do not agree at all”, “7=agree completely”)

- Please specify, on the answer sheet under section D, which did you consider to be your culture of origin

Section E

1. Think about your closest friends, with whom you kept regular contact in the last months:

a. How many have the same, or a very similar, heritage culture as you?

b. How many have the same heritage culture as your companion?

c. How many have a distinct heritage culture from both?

2. Exclusively for foreign-born respondents. Answer to the following statements in a scale from “1=Strongly disagree” to “5=Strongly agree”:

a. I frequently feel troubled understanding some of the Italian ways of interaction

b. It makes me feel good to adapt to the Italian social norms

c. I would rather behave as I was taught within my heritage culture than in the Italian way

d. It comes as a relief for me when I get to interact with someone sharing my heritage culture

Section F

1. Exclusively for foreign-born respondents. Focusing on the initial period adapting to the Italian culture, answer to the following statements in a scale from “1=Strongly disagree” to “5=Strongly agree”:

a. I frequently felt looked at with suspicion by Italian strangers

b. Actions that in my heritage culture would be considered normal were often judged with disapproval

c. Due to cultural differences, I sometimes had a sense of exclusion in social contexts

d. I occasionally felt a need to adapt to some of the Italian social norms in order to prevent negative reactions from occurring

How well did you understand the questions in this survey? (“1=barely” to “7=completely”)
