# Supplementary material for: Examining acculturation in mixed-couples to test cultural transmission mechanisms
Source: PLoS One. 2022 Apr 6;17(4):e0266229. doi: 10.1371/journal.pone.0266229 (PMC8985958; doi:10.1371/journal.pone.0266229)
Supplement: S2 Text — (PDF) [file pone.0266229.s017.pdf]

Examining acculturation in mixed couples to test the external validity of factors from cultural transmission models - Portuguese version

Para participar neste projecto de investigação deverá corresponder a um dos seguintes requisitos:

1. Ser nascido e criado fora de Portugal, segundo uma cultura diversa. Ter um relacionamento e viver com alguém que cumpra o outro requisito;

2. Ser nascido e criado em Portugal, por pais Portugueses. Ter um relacionamento e viver com alguém que cumpra o outro requisito.

- Caso ambos os membros do casal queiram participar, cada um deve preencher um questionário individualmente -

Estima-se que responder a este questionário não devera demorar mais de 20 minutos. A participação é anónima: o seu nome ou qualquer contacto pessoal não serão registados; nem qualquer outra informação que permitam identificá-lo. Todos os dados serão tratados confidencialmente. São-nos de interesse apenas no seu todo.

Por favor leia as questões com cuidado e responda da forma mais precisa possível. Não existem respostas "certas" ou "erradas" - apenas nos é importante saber a sua opinião pessoal. Pedimos ainda que tente responder num quadro global, em vez de se focar demasiado no presente.

Aconselhamos também a leitura das "dúvidas comuns" no início de cada secção. Tal pode ser uma ajuda para um preenchimento mais rápido.

/

To participate in this survey, you must meet one of the following conditions:

1. Be born and raised outside of Portugal, within a different culture. Be in a relationship and living together with someone that meets the next condition;

2. Be born in and raised in Portugal, by Portuguese parents. Be in a relationship and living together with someone that meets the previous condition.

- In case both members of the couple want to participare, each one should fill a questionnaire -

Answering the survey should take no more than 20 minutes. The survey is anonymous: your name or any personal contact won’t be registered, nor any other information that could make you identifiable. All data are treated confidentially. They are of interest to us only as a group.

Please read each question carefully and answer as precisely as you can. There are no "right" or "wrong" answers – we are only interested in your personal opinion. Please try to keep in mind an overall picture, rather than focus too much on the present.

In addition we kindly advise the participants to read the "most frequent doubts" at the beggining of each section. It may be a great help to proceed quicker.

*Obrigatório

1. **Endereço de email** *

____

University of Kent | School of Anthropology and Conservation

Dúvidas frequentes / Most frequent doubts:

a) “Cultura(s) a que sente pertencer” = resposta livre; “Cultura herdada” = aquela de base, adquirida na educação durante a infância (não aquela do companheiro); “Cultura de origem” = aquela predominante dos pais.

b) Secção A, pergunta 3: "Anos de instrução"/”Years of education” entende-se o número total de anos da escola primária até ao último título de estudo (diploma, licenciatura, mestrado, ...).

c) Secção A, pergunta 6: os participantes Portugueses deverão escrever o número de meses passados no país de origem do companheiro (ou países com uma cultura muito semelhante). Os participantes que não são Portugueses deverão escrever a idade de chegada e o número de anos que viveram em Portugal.

d) Secção A, pergunta 13: refere-se à família da pessoa que está a preencher o questionário.

e) Secção A, pergunta 14: deve indicar uma percentagem para a compreensão da língua e uma outra para o quão bem se consegue exprimir através dela.

---

a) "Cultures you feel belonging to" = free answer; "Heritage culture" = your base culture, the one you have been rased with (never the same one as your partner); "Culture of origine" = the predominant one from your parents.

b) Section A, question 3: "Years of education" asks you for sum of the years you studied starting at primary school.

c) Section A, question 6: Portuguese participants should write the number of months spent in the companion's country of origin (or countries with a very similar culture). Non Portuguese participants, on their turn, should write the age of arrival and also the number of years spent in Portugal.

d) Section A, question 14: you should indicate a percentage for the language comprehension, and another for how well do you can express yourself through it.

Secção A / Section A

Neste secção encontrará algumas breves perguntas de carácter demográfico / In this section some brief demographic questions will be asked

2. **Sexo / Sex** *

3. **Idade / Age** *

4. **Anos de instrução / Years of education** *

5. **Trabalhou nos últimos 6 meses? / Have you been employed in the last 6 months?** *
Marcar apenas uma oval.

- Sim / Yes
- Não / No

6. **Com a disponibilidade económica do seu agregado... ("1=vive-se com muita dificuldade", "2=vive-se com dificuldade", "3=dá para viver", "4=vive-se de modo confortável", "5=vive-se de modo muito confortável") / With your present household income... ("1=is very difficult to live", "2=is difficult to live", "3=we are coping", "4=we are living comfortably", "5=we are living very comfortably")** *
Marcar apenas uma oval.

	1	2	3	4	5	
vive-se com muita dificuldade / is very difficult to live						vive-se de modo muito confortável / we are living very comfortably

7. **Nasceu em Portugal? (A) Caso sim: número total de meses passados no país de origem do seu companheiro, ou num outro com uma cultura similar; (B) Caso não: i) idade de chegada a Portugal, ii) número de anos vivos em Portugal / Born in Portugal? (A) If yes: total number of months spent in your companion's country of origin or countries with a similar culture; (B) If no: i) age of arrival in Portugal, ii) number of years spent living in Portugal** *

8. **Nos últimos 10 anos em que cidade portuguesa habitou mais tempo? / Within the last 10 years, in which portuguese city did you spent most time living?** *

9. **Desde há quanto tempo dura a vossa relação? / For how long have you and your companion been together?** *

10. **É casado com o seu companheiro actual? / Are you married with your current partner?** *
Marcar apenas uma oval.

- Sim / Yes
- Não / No

11. **Esteve casado com alguém originário do mesmo país do seu companheiro? / Have you been formerly married with someone from the same country of origin as your companion?** *
Marcar apenas uma oval.

- Sim / Yes
- Não / No

12. **Quantos filhos tem? / How many children have you had?** *

13. **Quantos filhos teve com o seu actual companheiro? / How many children have you had with your actual companion?** *

14. **Numa escala de "1=muito má" a "5=muito boa" com "0=não mantenho contacto com eles", como descreveria a sua relação geral com os seus pais e restante família próxima? / In a scale from "1=Very bad" to "5=Very good" with "0=I don't maintain contact with them", how would you describe your overall relationship with your parents and close family?** *

Marcar apenas uma oval.

	0	1	2	3	4	5	
Não mantenho contacto com eles / I don't maintain contact with them	⬭	⬭	⬭	⬭	⬭	⬭	Muito boa / Very good

15. **Relativamente à língua materna do seu companheiro (respondendo com uma percentagem, %): i) Quão bem compreende a língua falada? ii) Quão bem se consegue exprimir através dela? / Regarding your companion's maternal language (answer both using percentages, %): i) How much of it can you understand when spoken? ii) How much can you produce when you want to express through it?** *

16. **Declare a qual ou a quais cultura(s) sente pertencer (ou escreva "nenhuma" se necessário) / Please state which culture(s) do you feel belonging to (or write "none" if that is the case)** *

Secção B / Section B

Muitas destas perguntas referir-se-ão à sua "cultura herdada", aquela que o influenciou mais (além da cultura do seu parceiro). Pode ser a cultura de nascimento, a cultura em que foi educado ou uma outra que faça parte da sua vivência. Se houverem várias culturas, por favor escolha aquela que o influenciou mais (ex. Irlandesa, Chinesa, Mexicana). Se sentir que não foi influenciado por nenhuma cultura além daquela do seu companheiro, por favor tente identificar uma cultura que poderá ter tido impacto nas gerações passadas da sua família. Responda usando a seguinte escala:

1 2 3 4 5 6 7 8
9
Discordo plenamente Discordo Neutral Concordo

Concordo plenamente

/

Many of these questions will referrer to your “heritage culture”, meaning the culture that influenced you most (other than your companion’s culture). It may be the culture of your birth, the culture in which you have been raised, or another culture that forms part of your background. If there are several of these cultures, please pick the one that has influenced you most (e.g. Irish, Chinese, Mexican). If you do not feel that you have been influenced by any other culture than your companions’ one, please try to identify a culture that may have had an impact on previous generations of your family. Answer in the following scale:

1	2	3	4	5	6	7	8	9
Strongly disagree		Disagree		Neutral Depends	Agree		Strongly	agree

Dúvidas frequentes / Most frequent doubts:

a) “Cultura(s) a que sente pertencer” = resposta livre; “Cultura herdada” = aquela de base, adquirida na educação durante a infância (não aquela do companheiro); “Cultura de origem” = aquela predominante dos pais.

---

a)"Cultures you feel belonging to" = free answer; "Heritage culture" = your base culture, the one you have been rased with (never the same one as your partner); "Culture of origine" = the predominant one from your parents.

17. **Frequentemente participo nas tradições da minha cultura herdada / I often participate in my heritage cultural traditions** *

Marcar apenas uma oval.

	1	2	3	4	5	6	7	8	9	
Discordo plenamente / Strongly disagree	◯	◯	◯	◯	◯	◯	◯	◯	◯	Concordo plenamente / Strongly agree

18. **Frequentemente participo nas tradições da cultura do meu companheiro / I often participate in my companion’s cultural traditions** *

Marcar apenas uma oval.

	1	2	3	4	5	6	7	8	9	
Discordo plenamente /Strongly disagree	◯	◯	◯	◯	◯	◯	◯	◯	◯	Concordo plenamente /Strongly agree

19. **Agrada-me desenvolver actividades sociais com pessoas com a minha cultura herdada / I enjoy social activities with people from the same heritage culture as myself** *

Marcar apenas uma oval.

	1	2	3	4	5	6	7	8	9	
Discordo plenamente / Strongly disagree	◯	◯	◯	◯	◯	◯	◯	◯	◯	Concordo plenamente / Strongly agree

20. **Agrada-me desenvolver actividades sociais com pessoas com a mesma cultura herdada que o meu companheiro / I enjoy social activities with people from the same heritage culture as my companion** *

Marcar apenas uma oval.

	1	2	3	4	5	6	7	8	9	
Discordo plenamente / Strongly disagree	◯	◯	◯	◯	◯	◯	◯	◯	◯	Concordo plenamente / Strongly agree

21. **Sinto-me confortável a trabalhar com pessoas da minha cultura herdada / I am comfortable working with people of the same heritage culture as myself** *

Marcar apenas uma oval.

	1	2	3	4	5	6	7	8	9	
Discordo plenamente / Strongly disagree	◯	◯	◯	◯	◯	◯	◯	◯	◯	Concordo plenamente / Strongly agree

22. **Sinto-me confortável a trabalhar com pessoas da cultura herdada do meu parceiro / I am comfortable working with people of the same heritage culture as my companion** *

Marcar apenas uma oval.

	1	2	3	4	5	6	7	8	9	
Discordo plenamente / Strongly disagree	◯	◯	◯	◯	◯	◯	◯	◯	◯	Concordo plenamente / Strongly agree

23. **Agradam-me as actividades de lazer (filmes, música, etc...) da minha cultura herdada / I enjoy entertainment (movies, music, etc…) from my heritage culture** *

Marcar apenas uma oval.

	1	2	3	4	5	6	7	8	9	
Discordo plenamente / Strongly disagree	◯	◯	◯	◯	◯	◯	◯	◯	◯	Concordo plenamente / Strongly agree

24. **Agradam-me as actividades de lazer (filmes, música, etc...) da cultura do meu companheiro / I enjoy entertainment (movies, music, etc…) from my companion's culture** *

Marcar apenas uma oval.

	1	2	3	4	5	6	7	8	9	
Discordo plenamente / Strongly disagree	◯	◯	◯	◯	◯	◯	◯	◯	◯	Concordo plenamente / Strongly agree

25. **Frequentemente assumo comportamentos típicos da minha cultura herdada / I often behave in ways that are typical to my heritage culture** *
Marcar apenas uma oval.

	1	2	3	4	5	6	7	8	9	
Discordo plenamente / Strongly disagree										Concordo plenamente / Strongly agree

26. **Frequentemente assumo comportamentos típicos da cultura do meu companheiro / I often behave in ways that are typical to my companion's culture** *
Marcar apenas uma oval.

	1	2	3	4	5	6	7	8	9	
Discordo plenamente / Strongly disagree										Concordo plenamente / Strongly agree

27. **Para mim é importante manter ou desenvolver práticas da minha cultura herdada / It is important to me to maintain or develop the practices of my heritage culture** *
Marcar apenas uma oval.

	1	2	3	4	5	6	7	8	9	
Discordo plenamente / Strongly disagree										Concordo plenamente / Strongly agree

28. **Para mim é importante manter ou desenvolver práticas da cultura do meu companheiro / It is important to me to maintain or develop practices of my companion's culture** *
Marcar apenas uma oval.

	1	2	3	4	5	6	7	8	9	
Discordo plenamente / Strongly disagree										Concordo plenamente / Strongly agree

29. **Acredito nos valores da minha cultura herdada / I believe in the values of my heritage culture** *
Marcar apenas uma oval.

	1	2	3	4	5	6	7	8	9	
Discordo plenamente / Strongly disagree										Concordo plenamente / Strongly agree

30. **Acredito nos valores tradicionais da cultura do meu companheiro / I believe in the mainstream values from my companion's culture** *

Marcar apenas uma oval.

	1	2	3	4	5	6	7	8	9	
Discordo plenamente / Strongly disagree	⬭	⬭	⬭	⬭	⬭	⬭	⬭	⬭	⬭	Concordo plenamente / Strongly agree

31. **As piadas e o humor da minha cultura herdada divertem-me / I enjoy the jokes and humour of my heritage culture** *

Marcar apenas uma oval.

	1	2	3	4	5	6	7	8	9	
Discordo plenamente / Strongly disagree	⬭	⬭	⬭	⬭	⬭	⬭	⬭	⬭	⬭	Concordo plenamente / Strongly agree

32. **As piadas e o humor da cultura do meu companheiro divertem-me / I enjoy typical jokes and humour from my companion's culture** *

Marcar apenas uma oval.

	1	2	3	4	5	6	7	8	9	
Discordo plenamente / Strongly disagree	⬭	⬭	⬭	⬭	⬭	⬭	⬭	⬭	⬭	Concordo plenamente / Strongly agree

33. **Interessa-me ter amigos da minha cultura herdada / I am interested in having friends from my heritage culture** *

Marcar apenas uma oval.

	1	2	3	4	5	6	7	8	9	
Discordo plenamente / Strongly disagree	⬭	⬭	⬭	⬭	⬭	⬭	⬭	⬭	⬭	Concordo plenamente / Strongly agree

34. **Interessa-me ter amigos da cultura do meu companheiro / I am interested in having friends from my companion's culture** *

Marcar apenas uma oval.

	1	2	3	4	5	6	7	8	9	
Discordo plenamente / Strongly disagree	⬭	⬭	⬭	⬭	⬭	⬭	⬭	⬭	⬭	Concordo plenamente / Strongly agree

35. **Por favor especifique qual considera ser a sua cultura herdada / Please specify which did you consider to be your heritage culture** *

Secção C / Section C

Os seguintes itens referem-se à sua percepção da qualidade da relação que tem com o seu companheiro / The following items refer to your perceptions of the relationship you have with your companion.

Dúvidas frequentes / Most frequent doubts:

a) (versão inglesa) "Dependable" não significa "dependent", tem sim um significado próximo a "fiável".

b) (ambas as versões) Algumas perguntas são muito semelhantes, mas não devem fazer caso, é mesmo assim.

---

a) "Dependable" means "fiável", not "dependant".

b) Some questions are very similar, but you don't need to focus on that. No need to worries.

36. **Quão satisfeito se sente com a sua relação? / How satisfied are you with your relationship?** *

Marcar apenas uma oval.

	1	2	3	4	5	6	7	
Nada / Not at all	◯	◯	◯	◯	◯	◯	◯	Extremamente / Extremely

37. **Quão contente se sente com a sua relação? / How content are you with your relationship?** *

Marcar apenas uma oval.

	1	2	3	4	5	6	7	
Nada / Not at all	◯	◯	◯	◯	◯	◯	◯	Extremamente / Extremely

38. **Quão feliz se sente com a sua relação? / How happy are you with your relationship?** *

Marcar apenas uma oval.

	1	2	3	4	5	6	7	
Nada / Not at all	◯	◯	◯	◯	◯	◯	◯	Extremamente / Extremely

39. **Quão impenhado está na sua relação? / How committed are you to your relationship?** *

Marcar apenas uma oval.

	1	2	3	4	5	6	7	
Nada / Not at all	⬭	⬭	⬭	⬭	⬭	⬭	⬭	Extremamente / Extremely

40. **Quão dedicado é à sua relação? / How dedicated are you to your relationship?** *

Marcar apenas uma oval.

	1	2	3	4	5	6	7	
Nada / Not at all	⬭	⬭	⬭	⬭	⬭	⬭	⬭	Extremamente / Extremely

41. **Quão devoto é à sua relação? / How devoted are you to your relationship?** *

Marcar apenas uma oval.

	1	2	3	4	5	6	7	
Nada / Not at all	⬭	⬭	⬭	⬭	⬭	⬭	⬭	Extremamente / Extremely

42. **Quão íntima é a sua relação? / How intimate is your relationship?** *

Marcar apenas uma oval.

	1	2	3	4	5	6	7	
Nada / Not at all	⬭	⬭	⬭	⬭	⬭	⬭	⬭	Extremamente / Extremely

43. **Quão próxima é a sua relação? / How close is your relationship?** *

Marcar apenas uma oval.

	1	2	3	4	5	6	7	
Nada / Not at all	⬭	⬭	⬭	⬭	⬭	⬭	⬭	Extremamente / Extremely

44. **Quão se sente ligado ao seu companheiro? / How connected are you to your partner?** *

Marcar apenas uma oval.

	1	2	3	4	5	6	7	
Nada / Not at all	⬭	⬭	⬭	⬭	⬭	⬭	⬭	Extremamente / Extremely

45. **Quão confia no seu companheiro? / How much do you trust your partner?** *

Marcar apenas uma oval.

	1	2	3	4	5	6	7	
Nada / Not at all	⬭	⬭	⬭	⬭	⬭	⬭	⬭	Extremamente / Extremely

46. **Quão pode contar com o seu companheiro? / How much can you count on your partner?** *

Marcar apenas uma oval.

	1	2	3	4	5	6	7	
Nada / Not at all	◯	◯	◯	◯	◯	◯	◯	Muitíssimo / Extremely

47. **Quão fiável é o seu parceiro? / How dependable is your partner?** *

Marcar apenas uma oval.

	1	2	3	4	5	6	7	
Nada / Not at all	◯	◯	◯	◯	◯	◯	◯	Extremamente / Extremely

48. **Quanto ama o seu companheiro? / How much do you love your partner?** *

Marcar apenas uma oval.

	1	2	3	4	5	6	7	
Nada / Not at all	◯	◯	◯	◯	◯	◯	◯	Muitíssimo / Extremely

49. **Quanto adora o seu parceiro? / How much do you adore your partner?** *

Marcar apenas uma oval.

	1	2	3	4	5	6	7	
Nada / Not at all	◯	◯	◯	◯	◯	◯	◯	Muitíssimo / Extremely

50. **Quanto preza o seu companheiro? / How much do you cherish your partner?** *

Marcar apenas uma oval.

	1	2	3	4	5	6	7	
Nada / Not at all	◯	◯	◯	◯	◯	◯	◯	Extremamente / Extremely

Secção D / Section D

Esta secção refere-se a aspectos referentes à sua cultura de origem - a cultura predominante dos seus pais. Portanto, "família" aqui refere-se a eles e aos parentes mais próximos. As pessoas que partilham esta cultura mas que não fazem parte da sua família serão referidas como "conterrâneos". / This section refers to attitudes towards your culture of origin – the predominant culture endorsed by your parents. So, "family" here refers to them and other close kin. People sharing this culture but that are not kin will be referred as "countrymen".

Dúvidas frequentes / Most frequent doubts:

a) (ambas as versões) Algumas perguntas são muito semelhantes, mas não devem fazer caso, é mesmo assim.

b) "Cultura(s) a que sente pertencer" = resposta livre; "Cultura herdada" = aquela de base, adquirida na educação durante a infância (não aquela do companheiro); "Cultura de origem" = aquela predominante dos pais.

a) Some questions are very similar, but you don't need to focus on that. No need to worries.

b)"Cultures you feel belonging to" = free answer; "Heritage culture" = your base culture, the one you have been rased with (never the same one as your partner); "Culture of origine" = the predominant one from your parents.

51. **Quão importantes são para si os valores e as normas da sua cultura de origem - as ideias a respeito do modo certo de viver, as convicções acerca do que é apropriado e do que não é? ("0=nada importantes; "7=muito importantes") / How important to you are the values and norms of your culture of origin —its ideas about the right way to live, its beliefs about what is proper and what not? ("0=not important at all", "7=very important")** *

Marcar apenas uma oval.

0	1	2	3	4	5	6	7
◯	◯	◯	◯	◯	◯	◯	◯

52. **Deseja que a sua cultura de origem se mantenha viva na geração dos seus (presentes ou futuros) filhos? ("0=não me interessa minimamente"; "7=espero muitíssimo que sim") / Do you wish your culture of origin to be kept alive in the generation of your (present or future) children? ("0=I do not care at all", "7=I wish this very much")** *

Marcar apenas uma oval.

0	1	2	3	4	5	6	7
◯	◯	◯	◯	◯	◯	◯	◯

53. **Seria para si importante manter a cultura de origem da sua família e transmiti-la aos seus filhos? ("0=nada importante"; "7=muito importante") / Would it be important for you to maintain your family's culture of origin and pass it on to your children? ("0=not important at all", "7=very important")** *

Marcar apenas uma oval.

0	1	2	3	4	5	6	7
◯	◯	◯	◯	◯	◯	◯	◯

54. **Lamentaria se os seus (presentes ou futuros) filhos esquecessem ou perdessem a língua que os seus pais falavam? ("0=não me importaria"; "7=lamentaria muito") / Would you feel sorry if your (present or future) children would forget or lose the language your parents spoke? ("0=I would not mind", "7=I would feel very sorry")** *

Marcar apenas uma oval.

0	1	2	3	4	5	6	7
◯	◯	◯	◯	◯	◯	◯	◯

55. **Lamentaria se os seus (presentes ou futuros) filhos virassem as costas à religião da sua família/à visão não religiosa do mundo da sua família? ("0=não me importaria"; "7=lamentaria muito") / Would you feel sorry if your (present or future) children would turn their back on the religion of your family/the non-religious worldview of your family? ("0=I would not mind", "7=I would feel very sorry")** *

Marcar apenas uma oval.

0	1	2	3	4	5	6	7
⬭	⬭	⬭	⬭	⬭	⬭	⬭	⬭

56. **Quanto lhe pesaria a perda das normas e dos valores da sua família? ("0=não me pesaria"; "7=pesar-me-ia muito") / How much would you regret the loss of norms and values of your family? ("0=I would not mind", "7=I would feel very sorry")** *

Marcar apenas uma oval.

0	1	2	3	4	5	6	7
⬭	⬭	⬭	⬭	⬭	⬭	⬭	⬭

57. **Uma pergunta hipotética: lamentaria se - por qualquer razão - a sua cultura de origem deixasse de existir daqui a 500 anos? ("0=não me importaria"; "7=lamentaria muito") / A hypothetical question: Would you feel sorry if—for whatever reason—your culture of origin would not exist anymore 500 years from now? ("0=I would not mind", "7=I would feel very sorry")** *

Marcar apenas uma oval.

0	1	2	3	4	5	6	7
⬭	⬭	⬭	⬭	⬭	⬭	⬭	⬭

58. **Como é que se sente quando a televisão, rádio ou jornais reportam qualquer coisa negativa a respeito do seu país ou da cultura de origem da sua família? ("0=não me importa nada"; "7=faz-me zangar muito") / How do you feel when something negative is reported on the television, radio or the newspapers about your family's country or culture of origin? ("0=I don't mind at all", "7=this makes me very angry")** *

Marcar apenas uma oval.

0	1	2	3	4	5	6	7
⬭	⬭	⬭	⬭	⬭	⬭	⬭	⬭

59. **Como é que se sente quando a televisão, rádio ou jornais reportam qualquer coisa positiva a respeito do seu país ou da cultura de origem da sua família? ("0=não me importa nada"; "7=deixa-me muito feliz") / How do you feel when something positive is reported on the television, radio, or the newspapers about your family's country or culture of origin? ("0=I don't mind at all", "7=this makes me very happy")** *

Marcar apenas uma oval.

0	1	2	3	4	5	6	7
⬭	⬭	⬭	⬭	⬭	⬭	⬭	⬭

60. **A minha família preferiria que eu me casasse com alguém da minha cultura de origem ("0=discordo plenamente"; "7= concordo plenamente") / My family would prefer that I married someone from my culture of origin ("0=do not agree at all, 7=agree completely")** *

Marcar apenas uma oval.

0	1	2	3	4	5	6	7
◯	◯	◯	◯	◯	◯	◯	◯

61. **Se um conterrâneo conhecido se encontra em dificuldade, eu devo ajudá-lo ("0=não concordo nada"; "7=concordo plenamente") / If a countryman acquaintance is in trouble, I should provide help ("0=do not agree at all", "7=agree completely")** *

Marcar apenas uma oval.

0	1	2	3	4	5	6	7
◯	◯	◯	◯	◯	◯	◯	◯

62. **Por favor especifique qual considera ser a sua cultura de origem / Please specify which did you consider to be your culture of origin** *

Secção E / Section E

Dúvidas frequentes / Most frequent doubts:

a) Secção E, pergunta 1: escolha um número adequado para cada categoria, sem pensar demasiado. POR FAVOR: um número, e não uma quantidade relativa.

a) Section E, question 1: choose a number for each category, without thinking too much. PLEASE: a number, not a relative quantity.

1. Pense nos seus amigos mais próximos, com os quais manteve contacto regular nos últimos meses: / Think about your closest friends, with whom you kept regular contact in the last months:

63. **a) Quantos são da sua mesma, ou uma muito similar, cultura herdada? / How many have the same, or a very similar, heritage culture as you?** *

64. **b) Quantos são da mesma cultura herdada que o seu companheiro? / How many have the same heritage culture as your companion?** *

65. **c) Quantos são de uma cultura herdada diferente de ambos? / How many have a distinct heritage culture from both?** *

2. EXCLUSIVAMENTE PARA PARTICIPANTES NASCIDOS NO ESTRANGEIRO. Responda às seguintes afirmações: / EXCLUSIVELY FOR FOREIGN-BORN RESPONDANTS. Answer to the following statements:

PARTICIPANTES NASCIDOS EM PORTUGAL, POR FAVOR NÃO RESPONDAM / PARTICIPANTS BORN IN PORTUGAL PLEASE DO NOT RESPOND

66. **a) Frequentemente sinto dificuldade em compreender alguns modos de interagir dos Portugueses / I frequently feel troubled understanding some of the Portuguese ways of interaction**

Marcar apenas uma oval.

	1	2	3	4	5	
Discordo plenamente / Strongly disagree	◯	◯	◯	◯	◯	Concordo plenamente / Strogly agree

67. **b) Faz-me sentir bem adaptar-me às normas sociais Portuguesas / It makes me feel good to adapt to the Portuguese social norms**

Marcar apenas uma oval.

	1	2	3	4	5	
Discordo plenamente / Strongly disagree	◯	◯	◯	◯	◯	Concordo plenamente / Strogly agree

68. **c) Preferiria agir segundo os ensinamentos da minha cultura herdada do que da forma Portuguesa / I would rather behave as I was taught within my heritage culture than in the Portuguese way**

Marcar apenas uma oval.

	1	2	3	4	5	
Discordo plenamente / Strongly disagree	◯	◯	◯	◯	◯	Concordo plenamente / Strogly agree

69. **d) Para mim é um alívio quando posso interagir com pessoas que partilham a minha cultura herdada / It comes as a relief for me when I get to interact with someone sharing my heritage culture**

Marcar apenas uma oval.

	1	2	3	4	5	
Discordo plenamente / Strongly disagree	◯	◯	◯	◯	◯	Concordo plenamente / Strogly agree

Secção F (exclusivamente para participantes nascidos no estrangeiro) / Section F (exclusively for foreign-born respondents)

PARTICIPANTES NASCIDOS EM PORTUGAL, POR FAVOR NÃO RESPONDAM / PARTICIPANTS BORN IN PORTUGAL PLEASE DO NOT RESPOND

Concentrando-se no seu primeiro período de adaptação à cultura Portuguesa, responda às seguintes afirmações: / Focusing on the initial period adapting to the Portuguese culture, answer to the following statements:

70. **a) Frequentemente senti-me observado com deconfiança por desconhecidos Portugueses / I frequently felt looked at with suspicion by Portuguese strangers**

Marcar apenas uma oval.

	1	2	3	4	5	
Discordo plenamente / Strongly disagree						Concordo plenamente / Strogly agree

71. **b) Acções que na minha cultura herdada seriam consideradas normais, por vezes eram julgadas com desaprovação / Actions that in my heritage culture would be considered normal were often judged with disapproval**

Marcar apenas uma oval.

	1	2	3	4	5	
Discordo plenamente / Strongly disagree						Concordo plenamente / Strogly agree

72. **c) Devido a diferenças culturais, por vezes senti-me excluído em contextos sociais / Due to cultural differences, I sometimes had a sense of exclusion in social contexts**

Marcar apenas uma oval.

	1	2	3	4	5	
Discordo plenamente / Strongly disagree						Concordo plenamente / Strogly agree

73. **d) Senti ocasionalmente uma necessidade de me adaptar a algumas das normas sociais Portuguesas de modo a prevenir possíveis reacções negativas / I occasionally felt a need to adapt to some of the Portuguese social norms in order to prevent negative reactions from occurring**

Marcar apenas uma oval.

	1	2	3	4	5	
Discordo plenamente / Strongly disagree						Concordo plenamente / Strogly agree

Secção final / Final section

74. **Quão bem compreendeu este questionário? / How well did you understand the questions in this survey?** *

Marcar apenas uma oval.

	1	2	3	4	5	6	7	
Praticamente nada / Barely	◯	◯	◯	◯	◯	◯	◯	Completamente / Completely

Privacy and ethics (disponível apenas em Inglês) - 27th February 2019

Participation is voluntary, nobody should feel constrained to take part in the present study.

This project is being organized by Bernardo Guerra Machado, masters student in Evolution and Human Behaviour, under the supervision of Dr Roger Giner-Sorolla, both from the University of Kent, United Kingdom. The University of Kent's School of Anthropology and Conservation is financing the research. The University's Ethics & Governance Office has approved the proposed procedures for data collection. There isn't any risk foreseen for participants. On the other hand, no direct advantages will be provided; we can only state that, by better understanding the cultural transmission processes, immigrant policies can better informed and developed.

As a university we use personally-identifiable information to conduct research, including to improve health, care and services. As a publicly-funded organisation, we have to ensure that it is in the public interest when we use personally-identifiable information from people who have agreed to take part in research. This means that when you agree to take part in a research study, we will use your data in the ways needed to conduct and analyse the research study. Your rights to access, change or move your information are limited, as we need to manage your information in specific ways in order for the research to be reliable and accurate. If you withdraw from the study, we will keep the information about you that we have already obtained. To safeguard your rights, we will use the minimum personally identifiable information possible.

The University Charter sets out that 'the objects of the University are to advance education and disseminate knowledge by teaching, scholarship and research for the public benefit' (paragraph 3).

Health and care research should serve the public interest, which means that we have to demonstrate that our research serves the interests of society as a whole. We do this by following the UK Policy Framework for Health and Social Care Research.

If you wish to raise a complaint on how we have handled your personal data, you can contact our Data Protection Officer who will investigate the matter. If you are not satisfied with our response or believe we are processing your personal data in a way that is not lawful you can complain to the Information Commissioner's Office (ICO).

The University of Kent's Data Protection Officer can be contacted at:
https://www.kent.ac.uk/infocompliance/dp/contact.html

Suggested wording adapted from: Health Research Authority –
https://www.hra.nhs.uk/planning-and-improving-research/policies-standards-legislation/dataprotection-and-information-governance/gdpr-guidance/templates/transparency-wordingpublic-sector/

Furthermore, for clarification on some doubt, withdraw requests, wish to express interest to know the study's results and findings, or any other issue the researcher can be directly contacted through e-mail: bpfg2@kent.ac.uk

If the study gets published, the data will be made available for other researches. No personal contacts or any other information that could be used to track the participants or link the responses to them will be provided. Neither any digital records with participants personal information are kept.

ARW/DN 08/06/17

University of Kent | School of Anthropology and Conservation

75. **Confirmo ter lido a folha informativa deste estudo, datada 27 de Fevereiro de 2019. Tive a oportunidade de analisar a informação e esclarecer as minhas dúvidas / I confirm I have read and understand the information sheet dated 27th February 2019 for the above study.I have had the opportunity to consider the information, ask questions and have had these answered satisfactorily** *

Marcar tudo o que for aplicável.

☐ Concordo / I agree

76. **Compreendi que a minha participação é voluntária e que tenho liberdade de desistir a qualquer momento sem ter de dar qualquer justificação (contactando bpfg2@kent.ac.uk) / I understand that my participation is voluntary and that I am free to withdraw at any time without giving any reason (by contacting bpfg2@kent.ac.uk)** *

Marcar tudo o que for aplicável.

☐ Concordo / I agree

77. **Compreendi que as minhas respostas serão anonimizadas. Dou permissão aos membros desta equipa de investigação para acederem às minhas respostas anonimizadas / I understand that my responses will be anonymised before analysis. I give permission for members of the research team to have access to my anonymised responses** *

Marcar tudo o que for aplicável.

☐ Concordo / I agree

78. **Concordo em participar neste projecto de investigação / I agree to take part in the above research project** *

Marcar tudo o que for aplicável.

☐ Concordo / I agree

79. **Se o seu companheiro preencheu também o questionário, por favor escreva o seu e-mail (assim poderemos emparelhar as respostas) / Please state your companion's e-mail, in case you both filled the questionnaire (so we can pair the responses)**

____

80. **Gostaria de receber os resultados deste estudo? / Would you like to receive this study's results?**

Marcar tudo o que for aplicável.

☐ Sim / Yes

Obrigado pelo apoio! / Thank you for the support!

Será enviada uma cópia das suas respostas por email para o endereço que forneceu

Com tecnologia
Google Forms
