# Supplementary material for: Examining acculturation in mixed-couples to test cultural transmission mechanisms
Source: PLoS One. 2022 Apr 6;17(4):e0266229. doi: 10.1371/journal.pone.0266229 (PMC8985958; doi:10.1371/journal.pone.0266229)
Supplement: S3 Text — (PDF) [file pone.0266229.s018.pdf]

S3 Text. The Italian version of the questionnaire.

Sezione A - In questa sezione troverà alcune brevi domande di carattere demografico:

1. Sesso

2. Età

3. Anni di istruzione

4. Ha lavorato negli ultimi 6 mesi?

5. Con la disponibilità economica complessiva di chi abita con lei... (“1=si vive con molta difficoltà”, “2=si vive con difficoltà”, “3=si riesce a vivere”, “4=si vive in modo confortevole”, “5=si vive in modo molto confortevole”)

6. Nato in Italia?

a. Se sì: numero totale di mesi trascorsi nel paese di origine del vostro compagno oppure in paesi con una cultura similare

b. Se no: i) età di arrivo in Italia; ii) numero di anni trascorsi in Italia

7. Negli ultimi 10 anni, in quale città italiana ha abitato per la maggior parte del tempo?

8. Da quanto tempo state insieme lei e il suo partner?

9. È sposato con il suo attuale partner?

10. È stato sposato con qualcuno originario dallo stesso paese del suo partner?

11. Quanti figli ha?

12. Quanti figli ha avuto dal suo attuale partner?

13. In una scala da “1=molto male” a “5=molto bene” con “0=non ho rapporti con loro”, come descriverebbe complessivamente la sua relazione con i suoi genitori e i componenti più prossimi della sua famiglia?

14. Relativamente alla lingua materna del suo compagno (rispondendo in percentuali, %):

a. Quanto comprende la lingua parlata?

b. Quanto riesce a esprimersi attraverso la forma parlata?

15. Dichiari a quale o a quali culture sente di appartenere (o scriva “nessuna” se necessario).

Sezione B - Molte di queste domande si riferiranno alla sua “cultura ereditata”, intendendo la cultura che più la ha influenzata (oltre alla cultura del suo partner). Può trattarsi della cultura di nascita, della cultura in cui è cresciuto oppure un’altra cultura che costituisca parte del suo vissuto. Se ne esistono molte, per favore selezioni quella che la ha influenzata di più (es. Irlandese, Cinese, Messicana). Se non sente di essere stato influenzato da nessuna cultura al di là di quella del suo partner, cerchi di identificare quella che potrebbe avere avuto un impatto sulla generazione precedente nella sua famiglia. Risponda alle seguenti questioni in una scala da “1= in forte disaccordo” a “9=in forte accordo”:

1. Spesso prendo parte alle tradizioni della mia cultura ereditata

2. Spesso prendo parte alle tradizioni della cultura del mio partner

3. Mi piace svolgere le attività sociali con persone con la mia stessa cultura ereditata

4. Mi piace svolgere attività sociali con persone con la stessa cultura ereditata del mio partner

5. Mi sento a mio agio lavorando con persone con la mia stessa cultura ereditata

6. Mi sento a mio agio lavorando con persone con la stessa cultura ereditata del mio partner

7. Mi piacciono le attività di svago (film, musica, ecc…) della mia cultura ereditata

8. Mi piacciono le attività di svago (film, musica, ecc…) della cultura ereditata del mio partner

9. Spesso assumo comportamenti tipici della mia cultura ereditata

10. Spesso assumo comportamenti tipici della cultura ereditata del mio partner

11. È importante per me mantenere o sviluppare abitudini della mia cultura ereditata

12. È importante per me mantenere o sviluppare abitudini della cultura ereditata del mio partner

13. Credo nei valori della mia cultura ereditata

14. Credo nei valori tradizionali della cultura ereditata del mio partner

15. Mi divertono le battute e l'umorismo della mia cultura ereditata

16. Mi divertono le battute e l’umorismo della cultura ereditata del mio partner

17. Mi interessa avere amici della mia cultura ereditata

18. Mi interessa avere amici della cultura del mio partner

- Specifichi ora, nel foglio delle riposte, sotto la sezione B, quale ha considerato essere la sua cultura ereditata.

Sezione C - Le seguenti voci si riferiscono alle sue percezioni della relazione che ha con il suo compagno. Rispondi in una scala da “1=per niente” a “7=moltissimo”

1. Quanto è soddisfatto della sua relazione?

2. Quanto è contento della sua relazione?

3. Quanto è felice della sua relazione?

4. Quanto è impegnato nella sua relazione?

5. Quanto è dedito alla sua relazione?

6. Quanto è devoto alla sua relazione?

7. Quanto è intima la sua relazione?

8. Quanto è stretta la sua relazione?

9. Quanto siete legati lei e il suo partner?

10. Quanto si fida del suo partner?

11. Quanto può contare sul suo partner?

12. Quanto è affidabile il suo partner?

13. Quanto ama il suo partner?

14. Quanto adora il suo partner?

15. Quanto apprezza il suo partner?

Sezione D - Questa sezione si riferisce agli aspetti riguardo alla sua cultura di origine - intendendo la cultura predominante dei suoi genitori. Quindi “famiglia” si riferirà a loro e ad altri parenti stretti. Persone che condividono questa cultura, ma che non sono parenti saranno indicati come “conterranei”.

1. Quanto sono importanti per lei i valori e le norme della sua cultura di origine – le idee riguardo al giusto modo di vivere, le convinzioni riguardo ciò che è appropriato e ciò che non lo è? (“0=per niente importante; “7=molto importante”)

2. Desidera che la sua cultura di origine si mantenga viva nella generazione dei suoi (presenti o futuri) figli? (“0=Non mi interessa minimamente”; “7=lo spero moltissimo”)

3. Sarebbe per lei importante mantenere la cultura di origine della sua famiglia e trasmetterla ai suoi figli? (“0=per niente importante”; “7=molto importante”)

4. Le dispiacerebbe se I suoi (presenti o futuri) figli dimenticassero o perdessero la lingua parlata dai suoi genitori? (“0=non mi importerebbe”; “7=mi dispiacerebbe molto”)

5. Le dispiacerebbe se i suoi (presenti o futuri) figli voltassero le spalle alla religione della sua famiglia/alla visione non-religiosa del mondo della sua famiglia? (“0=non mi importerebbe”; “7=mi dispiacerebbe molto”)

6. Quanto rimpiangerebbe la perdita delle norme e dei valori della sua famiglia? (“0=non mi importerebbe”; “7=mi dispiacerebbe molto”)

7. Una domanda ipotetica: Si dispiacerebbe se -per qualunque ragione- la sua cultura di origine non esistesse più tra 500 anni? (“0=non mi importerebbe”; “7=mi dispiacerebbe molto”)

8. Come si sente quando la televisione, la radio o i giornali riportano qualcosa di negativo riguardo al paese o alla cultura di origine della sua famiglia? (“0=non mi importa per niente”; “7=mi fa arrabbiare molto”)

9. Come si sente quando la televisione, la radio o i giornali riportano qualcosa di positivo riguardo al paese o alla cultura di origine della sua famiglia? (“0=non mi importa per niente”; “7=mi rende molto felice”)

10. La mia famiglia preferirebbe che io sposassi qualcuno con la mia stessa cultura di origine (“0=non concordo per niente”; “7= concordo pienamente”)

11. Se un conterraneo conoscente si trova in difficoltà, sento di dover aiutare (“0=non concordo per niente”; “7=concordo pienamente”)

- Specifichi ora, nel foglio delle risposte, sotto la sezione D, quale ha considerato essere la sua cultura di origine.

Sezione E

1. Pensi ai suoi amici più cari, quelli con cui ha mantenuto regolari contatti negli ultimi mesi:

a. Quanti hanno la sua stessa cultura ereditata (o una molto simile)?

b. Quanti hanno la stessa cultura ereditata del suo partner?

c. Quanti hanno una cultura ereditata differente da entrambi?

2. Esclusivamente per I partecipanti nati all'estero. Risponda alle seguenti affermazioni in una scala da "1=in forte disaccordo" a "5=in forte accordo":

a. Frequentemente ho difficoltà nel comprendere alcuni modi di interagire degli Italiani

b. Mi fa sentire bene adattarmi alle norme sociali Italiane

c. Preferirei agire secondo la mia cultura ereditata invece che secondo la maniera italiana

d. Mi dà sollievo interagire con persone che condividono la mia stessa cultura ereditata

Sezione F

1. Esclusivamente per i partecipanti nati all’estero. Concentrandosi nel suo primo periodo di adatazione alla cultura Italiana, risponda alle seguenti affermazioni in una scala da “1= in forte disaccordo” a “5=in forte accordo”:

a. Molte volte mi sento osservato con diffidenza dagli italiani sconosciuti

b. Azioni che nella mia cultura ereditata sarebbero considerate normali a volte sono giudicate con disapprovazione nella cultura italiana

c. A causa di differenze di tipo culturale, a volte mi sono sentito escluso in alcuni contesti sociali

d. A volte sento che devo adattarmi ad alcune delle norme sociali italiane per evitare possibili reazioni negative

Quanto ha compreso le domande di questo sondaggio? (“1=a malapena”; “7=completamente”)
